# Novel 1-Amidino-4-Phenylpiperazines as Potent Agonists at Human TAAR1 Receptor: Rational Design, Synthesis, Biological Evaluation and Molecular Docking Studies

**DOI:** 10.3390/ph13110391

**Published:** 2020-11-14

**Authors:** Valeria Francesconi, Elena Cichero, Evgeny V. Kanov, Erik Laurini, Sabrina Pricl, Raul R. Gainetdinov, Michele Tonelli

**Affiliations:** 1 Dipartimento di Farmacia, Università degli Studi di Genova, Viale Benedetto XV, 3, 16132 Genova, Italy; francesconi.phd@difar.unige.it; 2 Institute of Translational Biomedicine, St. Petersburg State University, 199034 St. Petersburg, Russia; vonkanoff@gmail.com (E.V.K.); gainetdinov.raul@gmail.com (R.R.G.); 3 Molecular Biology and Nanotechnology Laboratory (MolBNL@UniTS), Department of Engineering and Architecture, University of Trieste, Piazzale Europa 1, 34127 Trieste, Italy; erik.laurini@dia.units.it (E.L.); sabrina.pricl@dia.units.it (S.P.); 4 Department of General Biophysics, Faculty of Biology and Environmental Protection, University of Lodz, 90-236 Lodz, Poland; 5 St. Petersburg University Hospital, St. Petersburg State University, 199034 St. Petersburg, Russia

**Keywords:** trace amine-associated receptor 1 (TAAR1), human TAAR1 agonists, 1-amidino-4-phenylpiperazines, pharmacophore model, docking studies

## Abstract

Targeting trace amine-associated receptor 1 (TAAR1) receptor continues to offer an intriguing opportunity to develop innovative therapies in different pharmacological settings. Pursuing our endeavors in the search for effective and safe human TAAR1 (*h*TAAR1) ligands, we synthesized a new series of 1-amidino-4-phenylpiperazine derivatives (**1**–**16**) based on the application of a combined pharmacophore model/scaffold simplification strategy for an in-house series of biguanide-based TAAR1 agonists. Most of the novel compounds proved to be more effective than their prototypes, showing nanomolar EC_50_ values in functional activity at *h*TAAR1 and low general cytotoxicity (CC_50_ > 80 µM) when tested on the Vero-76 cell line. In this new series, the main determinant for TAAR1 agonism ability appears to result from the appropriate combination between the steric size and position of the substituents on the phenyl ring rather than from their different electronic nature, since both electron-withdrawing and electron donor groups are permitted. In particular, the *ortho*-substitution seems to impose a more appropriate spatial geometry to the molecule that entails an enhanced TAAR1 potency profile, as experienced, in the following order, by compounds **15** (2,3-diCl, EC_50_ = 20 nM), **2** (2-CH_3,_ EC_50_ = 30 nM), **6** (2-OCH_3,_ EC_50_ = 93 nM) and **3** (2-Cl, EC_50_ = 160 nM). Apart from the interest in them as valuable leads for the development of promising *h*TAAR1 agonists, these simple small molecules have further allowed us to identify the minimal structural requirements for producing an efficient *h*TAAR1 targeting ability.

## 1. Introduction

Trace amine-associated receptor 1 (TAAR1) is increasingly being recognized as a druggable target in the treatment of several diseases, particularly for Central Nervous System (CNS) disorders [1,2]. TAAR1 is expressed in different regions of the brain and several peripheral tissues (e.g., pancreas, stomach, intestines, testes, and leukocytes) [3]. 

TAAR1 was identified as responsive to a class of endogenous ligands, called trace amines (TAs) [3]. In particular, TAs such as tyramine (TYR), β-phenylethylamine (β-PEA) and 3-iodothyronamine (T_1_AM) were identified as the most likely physiological, high-affinity TAAR1 agonists with potency in the nanomolar range; however, both ligands and receptor-specific functions still deserve further investigations to be clarified [4,5]. TAAR1 can also recognize classical monoamine neurotransmitters as endogenous ligands, which are endowed with a micromolar degree of efficacy in stimulating TAAR1 [3,5]. TAs and classical biogenic amine pathways significantly overlap, even if TAs are expressed at markedly lower levels in vivo.

Dysregulation of TAs is also considered as one of the contributing factors to the etiology of many diseases, including not only schizophrenia, depression, attention deficit hyperactivity disorder (ADHD), but also sleep disorders and metabolic syndrome [2,6,7]. This scenario is currently stimulating an increasing interest in specifically investigating the TAAR1 role in CNS, where receptor activation is associated with precise control of monoaminergic circuits. TAAR1 is able to regulate dopamine (DA) [8,9], 5-hydroxytriptamine (5-HT or serotonin) [10], and glutamate [11] neurotransmission, decreasing the basal firing rates and negatively modulating receptor sensitivity. In this context, the initial studies revolved around drug repurposing strategies, drawing inspiration from the T_1_AM scaffold and known dopaminergic, adrenergic and serotonergic ligands. Different structurally related synthetic compounds and psychostimulant drugs have been screened and developed as potent TAAR1 agonists [12]. However, due to the structural similarity with their prototypes, most of these small molecules continued to be affected by an impaired selectivity profile towards the TAAR1 receptor with respect to other, highly related G-protein-coupled receptors (GPCRs) (monoaminergic systems) [13]. By chemical manipulation of T_1_AM, Chiellini et al. developed a class of halogen-free diphenylmethane derivatives, which displayed both in vitro and in vivo efficacy [14,15]. Later analogues showed a potency similar or even superior to that of their prototypes as TAAR1 agonists; however, these molecules shared with T_1_AM some non-TAAR1-mediated functional effects (e.g., stimulation of hepatic gluconeogenesis), which require further investigation for an adequate evaluation of the selectivity issue [16,17].

**Figure 1:**
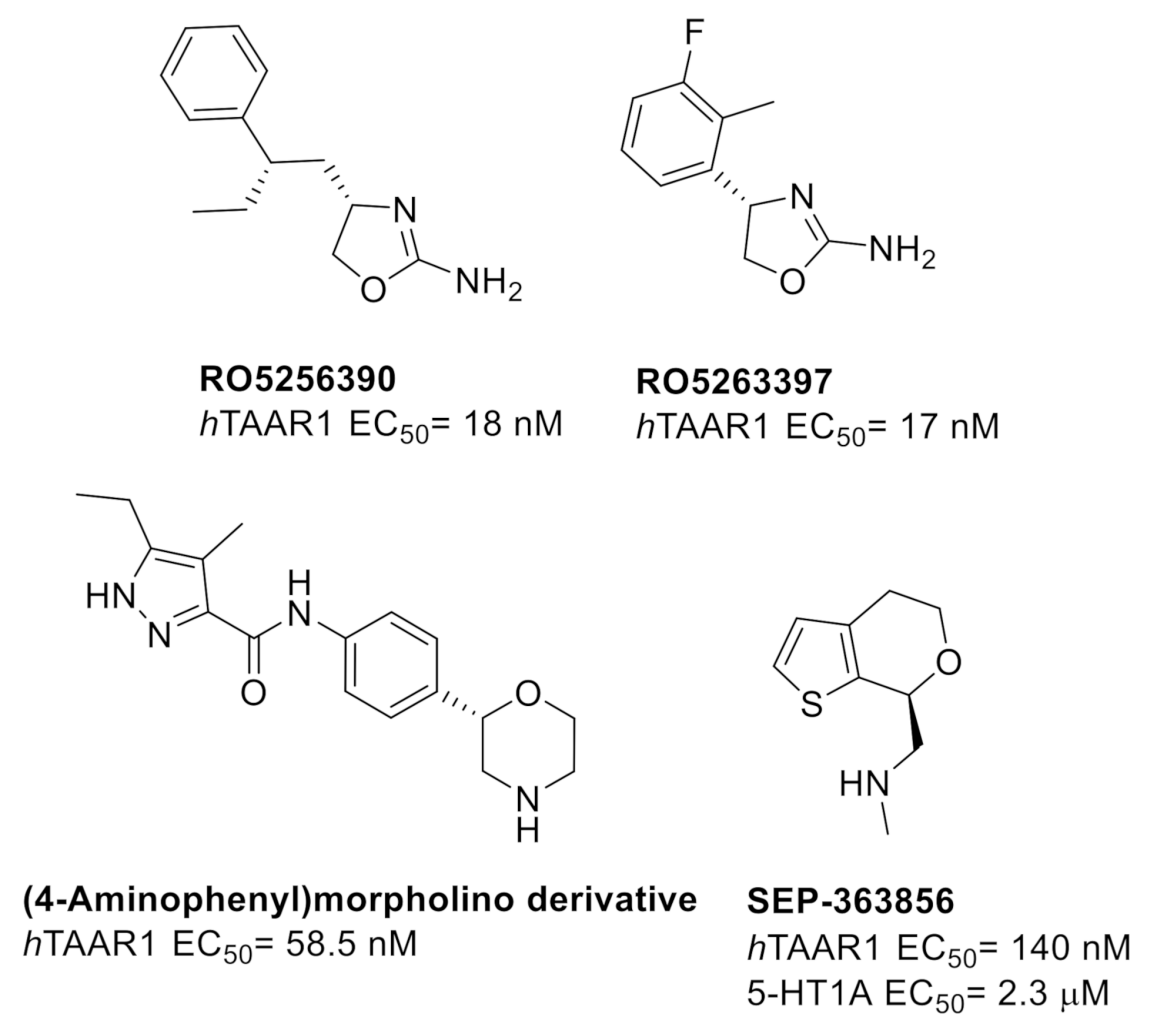
Chemical structure and functional activity of the most promising human trace amine-associated receptor 1 (*h*TAAR1) agonists.

Most efforts in the TAAR1 field were undertaken by the Hoffmann-La Roche pharmaceutical company, whose research focused on an iterative series of structural modifications of adrenergic medications; this led to the discovery of promising compounds (the so-called “RO family”), including the amino-oxazoline α_2A_-adrenergic receptor agonist S18616 [18]. A screening procedure involving over 100 target proteins allowed the assessment of high TAAR1 selectivity for these RO compounds, so they were widely used in functional assays to study the effects of TAAR1 stimulation. As a result, several potent full (e.g., RO5256390) [19] and partial TAAR1 agonists (e.g., RO5203648 and RO5263397, Figure 1) were obtained [20,21], which also showed an improved efficacy at *h*TAAR1.

The *h*TAAR1 agonists RO5256390 and RO5263397 also exhibited an addictive and synergistic effect in combination therapy with the atypical antipsychotic drug olanzapine (Zyprexa) [22], suggesting, for TAAR1 agonists, a potential antipsychotic-like activity along with a reduced risk of metabolic syndrome induced by the marketed drug. Hofmann-La Roche recently patented a 5-ethyl-4-methylpyrazole-3-carboxamide derivative based on the (2*S*)-(4-aminophenyl)morpholino scaffold as a first-in-class *h*TAAR1 partial agonist (EC_50_ = 58.5 nM, *h*TAAR1 efficacy 42%, Figure 1) for the treatment or prevention of psychiatric disorders [23]. Moreover, the selective TAAR1 agonists RO5166017 and RO5256390 were also proposed for the treatment of diabetes and obesity [24,25,26], being able to promote glucose-dependent insulin secretion in β-cells lines and human islets. In 2019, major progress in the quest for innovative antipsychotic medications was achieved by Sunovion Pharmaceuticals with the identification of the molecule SEP-363856 as a mixed TAAR1 and 5-HT1A agonist [27] (Figure 1); this compound is currently showing encouraging positive results in phase 2 and phase 3 clinical trials for the assessment of its efficacy and safety in patients with schizophrenia (ClinicalTrials.gov Identifier: NCT02969382, NCT04109950, NCT04325737) [28].

From the analysis of the different chemotypes thus far reported as TAAR1 agonists [1,3], a basic core and an aromatic/heteroaromatic moiety were identified as mandatory pharmacophore features of a TAAR1 ligand. Moreover, for a successful TAAR1 activity, these two required units need to be linked through a spacer of variable nature and length; this, in turn, modulates the overall molecular flexibility and, ultimately, the related TAAR1 binding ability of the relevant compounds.

**Figure 2:**
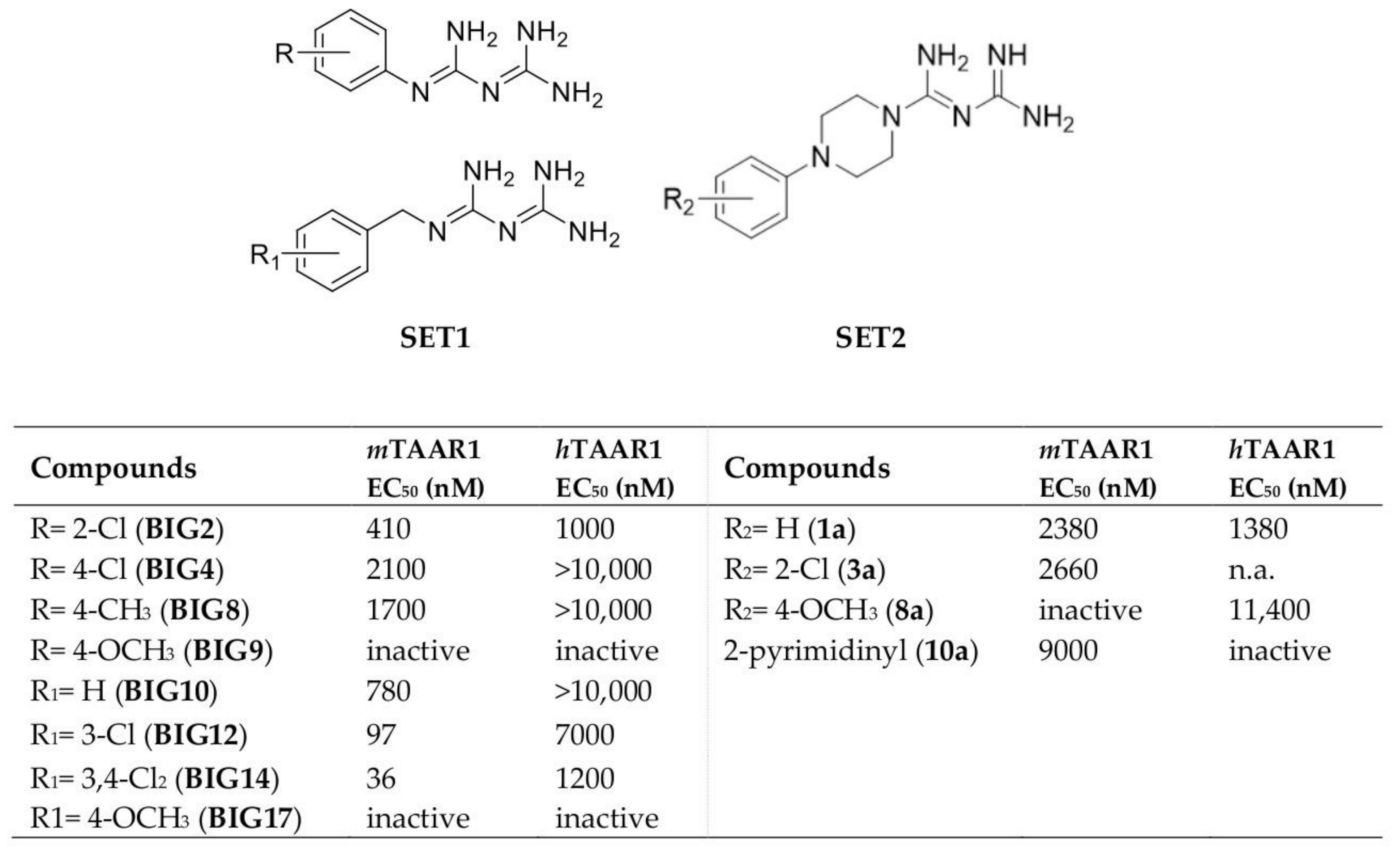
Chemical structure of previously studied biguanide-based derivatives as *m/h*TAAR1 agonists**.**

In this scenario, we started the study of new TAAR1 ligands, exploring phenyl or benzyl biguanides (SET1, Figure 2) as novel chemotypes [29] that share a selective murine and human TAAR1 agonism behavior with respect to murine TAAR5. In particular, the SET1 derivatives were endowed with higher specificity towards the mouse receptor (nanomolar range) with respect to the human orthologue (low micromolar range). Molecular docking studies at the *m*/*h*TAAR1 receptors enlightened the relevance of the ligand’s basic core in forming a key salt bridge with a conserved *m*/*h*TAAR1 D3.32 aspartic acid, and of the aromatic moiety in engaging π–π stacking and van der Waals contacts with a number of recurrent aromatic residues characterizing the receptor cavity [1,29,30].

In this context, we recently reported the development of two quantitative structure–activity relationship (QSAR) models exploring the agonism ability offered by different chemotypes towards murine and human TAAR1, including the aforementioned SET1 derivatives and the most potent agonists disclosed by Roche [31] (Supplementary data S1). The results allowed us to outline some species specificity preferences and to derive useful information for the synthesis of novel biguanide-based compounds (SET2, Figure 2) exhibiting selective agonism towards the two orthologues.

In particular, in order to evaluate the proper distance between the two moieties, we designed these new arylbiguanide analogues based on the piperazine ring as a bifunctional and rigid spacer tethering the aromatic core with the biguanide moiety. These SET2 compounds also showed agonistic activity at TAAR1, mostly with a mouse TAAR1 (*m*TAAR1) species-selective profile, with the exception of **8a**, which bound only the human orthologue, but with poor affinity (Figure 2) [31]. From a careful analysis of the two SET1 and SET2 biguanide-based series, the best substitution pattern consisted of lipophilic groups on the aromatic ring (Cl, F, Br, CF_3_, CH_3_), with the electron-withdrawing chlorine atom representing the best performing substituent; on the contrary, the presence of polar groups (OCH_3_) on the phenyl ring resulted in negative effects, sometimes abolishing the activity. Interestingly, a comparable trend was also reported for the most notable TAAR1 targeting chemotypes thus far developed [1].

## 2. Results and Discussion

### 2.1. Design of hTAAR1 Agonists

**Figure 3:**
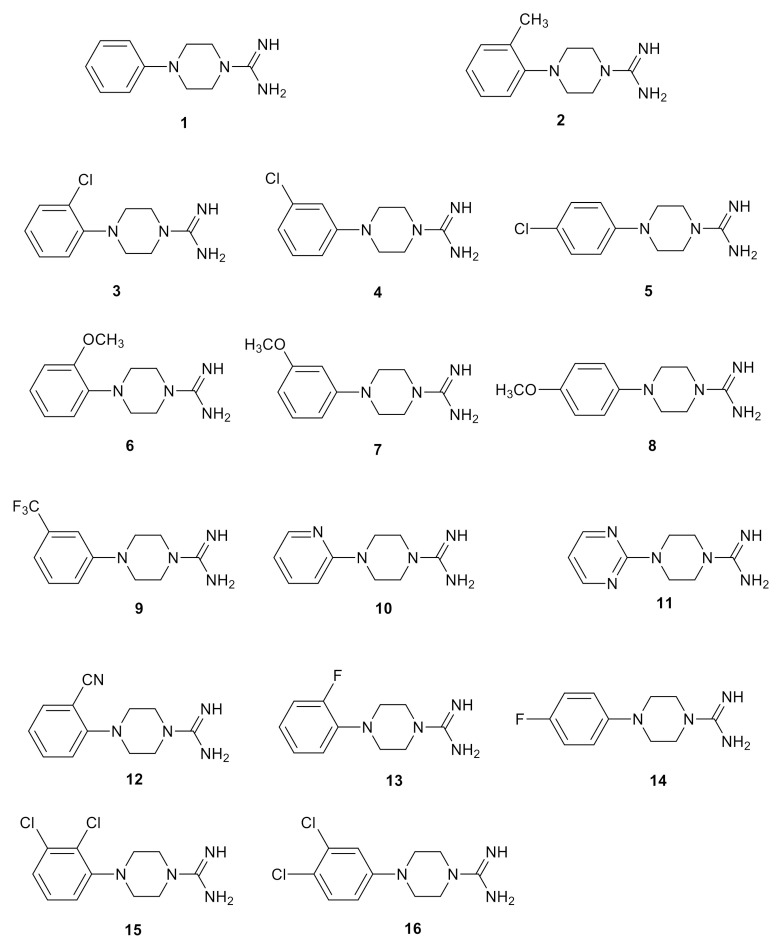
Chemical structures of the investigated *h*TAAR1 agonists.

In this work, we proceeded with the rational design of a new series of novel 1-amidino-4-phenylpiperazine derivatives (1–16) (Figure 3) based on information derived from the development of a preliminary pharmacophore model (PM), built by taking into account the most potent oxazolines thus far described (pEC_50_ values > 7.00 M) among those reported by Roche (see Table S1). This approach was combined with a scaffold simplification strategy for an in-house series of biguanide-based TAAR1 agonists (SET2) to probe the *h*TAAR1 targeting ability of this new series of amidine-containing derivatives.

We simplified the biguanide moiety of arylpiperazino-derived biguanides (SET2), which was replaced in favor of the simpler and flexible amidino group, with a view to improving the TAAR1 agonism ability. The aromatic ring of the novel 1-amidino-4-arylpiperazino scaffold was properly functionalized at different positions (**1**–**16**, Figure 3) introducing the more efficient lipophilic and electron-withdrawing substituents, but also including the polar and electron donor OCH_3_ group (**6**–**8**), in order to confirm the structure–activity relationship of the previously built SET1 and SET2 of biguanide-based TAAR1 agonists. To investigate the more suitable contribution for targeting the TAAR1 receptor, compounds **10** and **11** were decorated with electron-deficient rings (pyridine-2-yl and pyrimidin-2-yl, respectively) in place of their phenyl ring. These moieties were also included in different TAAR1 targeting scaffolds, being characterized by at least one electron-rich nitrogen, involved in a potential H-bond, or salt bridge interactions after protonation [1].

The bulk of the evidence in the literature [3,5,7] reports a fold decrease in affinity values and functional activity by using mouse and rat TAAR1 (*m/r*TAAR1) receptors as surrogate models of human orthologues (*h*TAAR1). In this regard, we limited the functional evaluation of the novel compounds only at *h*TAAR1, in order to obtain informative data; meanwhile, cytotoxicity assays were performed against Vero cells to explore the safety profile of these novel *h*TAAR1-targeting compounds. The molecular docking study explained the SAR of these compounds in relation to their binding mode to *h*TAAR1, revealing the key interaction for further improvements.

These combined studies have led to the identification of promising molecules worthy of further studies to assess their TAAR1 selectivity profile over monoaminergic GPCRs and to develop optimized TAAR1 ligands.

### 2.2. Pharmacophore Modeling

In our previous work, we reported the development of two quantitative structure–activity relationship (QSAR) models, exploring the agonism ability featured by different chemotypes toward murine and human TAAR1, including the in-house biguanide-based analogues (SET1) as well as the potent imidazolines and oxazolines published by Roche [31]. The relevant results allowed us to identify some species specificity preferences and to derive useful information for the synthesis of the aforementioned biguanides (SET2), which exhibited selective agonism towards the two orthologues.

Briefly, we focused on those compound chemical descriptors more specifically in relation to the human TAAR1 (model A), deciphering a limited number of key descriptors involved in efficient *h*TAAR1 targeting. Then, we assessed the role played by the same set of chosen descriptors in influencing agonistic activity at the murine receptor. In this way, a further QSAR model (model B) related to the murine orthologue agonism ability was built. 

Interestingly, model A unveils that seven out of the eight selected descriptors belong to the 3D class, mainly referring to area or volume measurements. These findings underscore the importance of the compound spatial conformation in influencing *h*TAAR1 activation, featuring a balanced hydrophobicity and hydrophilicity profile in tandem with a folded polar surface. Indeed, the unsubstituted 1-phenyl-4-biguanylpiperazine **1a** (*h*TAAR1 EC_50_ = 1380 nM, pEC_50_ = 5.86 M, Figure 2) was more potent than 1-benzylbiguanide (**BIG10**) (*h*TAAR1 EC_50_ > 10,000 nM). Smaller and conformationally locked compounds such as 1-phelylbiguanide (**BIG2**), **BIG4** and **BIG8** of the SET1 family proved to be all inactive as *h*TAAR1-targeting ligands, except for the 2-Cl substituted derivative **BIG2** (Figure 2). Conversely, the 3- and/or 4-substitution in this class of compounds led to more interesting derivatives when combined with the benzyl-based biguanides of SET1. On the whole, in SET1, these two structural architectures were mandatory to guarantee a proper conformer positioning within the receptor crevice. 

Herein, we proceeded with the development of a preliminary pharmacophore model considering the most potent oxazolines reported by Roche (pEC_50_ values > 7.00 M, Table S1), with the aim of gaining useful information about the most effective key features and reciprocal distances required in tailoring promising *h*TAAR1 ligands. This study was also conceived to provide an interesting computational tool to evaluate and guide the effectiveness of the simplification strategy, prior to the chemical synthesis and biological evaluation of new TAAR1 ligands. 

The derived pharmacophore model was generated using the pharmacophore consensus module integrated into the MOE software. The program is based on the identification and classification of the most commonly shared recurrent pharmacophore features within the proposed set of molecules. Any pharmacophore group is classified by an identification code associated with the program (ID), the percentage by which this feature appears among the molecules considered (SCORE), by a radius that exemplifies the maximum space within which this functional group can be placed within the ligand (RADIUS), and by a symbol that represents its role in terms of interaction with the receptor (EXPRESSION). Among these, aromatic rings and hydrophobic substituents at the ligands were identified as Aro and Hyd features, while H-bonding groups were described as Don or Acc when referring to donor or acceptor moieties included in the agonists. 

**Figure 4:**
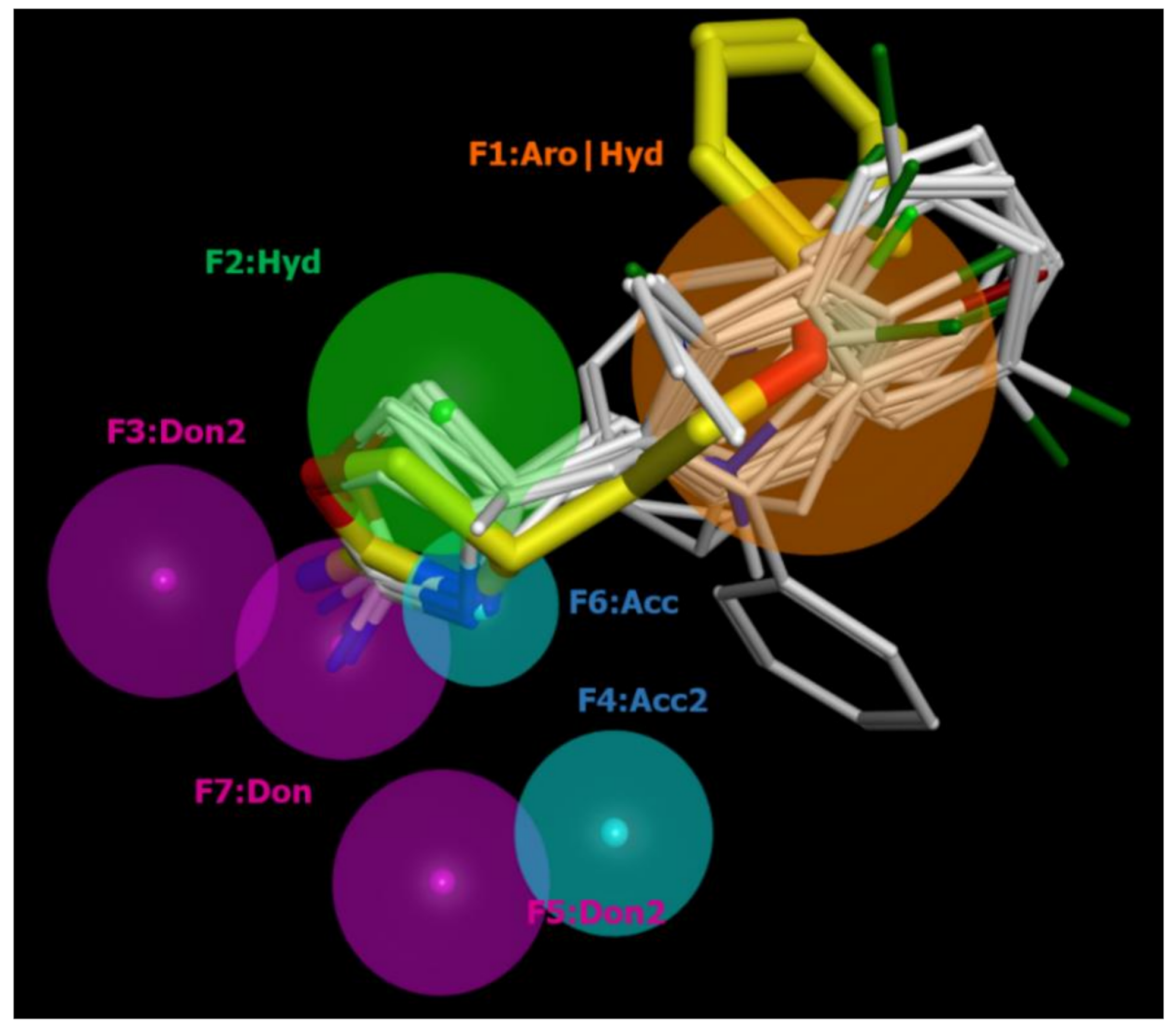
Alignment of the oxazolines included in the pharmacophore model generation (compounds **4b**, **5b**, **9b**–**11b**, **16b**, **18b**, **20b**–**23b**, **25b**, **29b**, **33b**–**37b**, Table S1), based on the chemical structure, as represented by the previous conformer analysis [31]. All oxazolines are shown as thin sticks, with the following atom color code: *C*, gray; halogen, green; *N*, blue; *O*, red). The reference compound **20b** is depicted as bold sticks with the same atom color code except for *C*, here in yellow for better visualization.

The alignment of the oxazolines **4b**, **5b**, **9b**–**11b**, **16b**, **18b**, **20b**–**23b**, **25b**, **29b**, **33b**–**37b** (Table S1, pEC_50_ values > 7.00 M) is shown in Figure 4, taking the most potent TAAR1 agonist **20b** (pEC_50_ = 8.05) as the reference compound.

Based on the data obtained, the most important pharmacophore requirements (represented by at least 80% of the molecules under examination) to design a *h*TAAR1 agonist include seven characteristic groups, especially H-bonding features properly tethered to at least two (hetero)aromatic or hydrophobic groups, as shown in Table 1.

In particular, Figure 4 shows a bulky aromatic ring (namely F1 Aro|Hyd) properly connected to a further hydrophobic core (F2:Hyd) positioning H-bonding features (such as F3:Don2, F4:Acc2, F5:Don2) in proximity of the corresponding principal F6:Acc and F7:Don (shared by all the most potent oxazolines). Interestingly, this information agrees with our previous QSARs, supporting a limited number of positively charged atoms in TAAR1 ligands engaged in a salt bridge with a conserved aspartic acid (D103) of the receptor.

**Figure 5:**
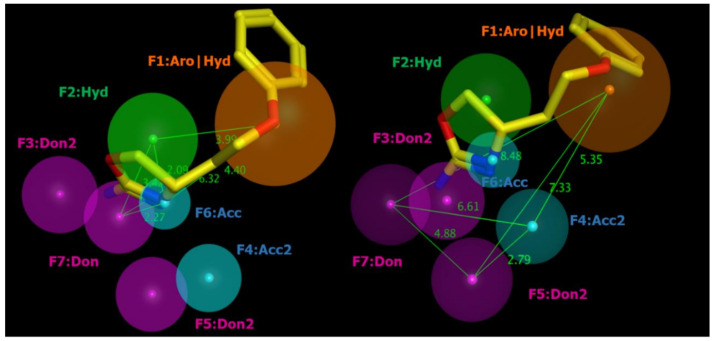
Pharmacophore features shared by most of the oxazolines acting as *h*TAAR1 agonists (Table S1). The most potent derivative **20b** is mapped onto the corresponding pharmacophore model (PM) features and is depicted by sticks (colors as in Figure 4). Distances between the main groups are shown as green lines and labelled (Å).

The expected reciprocal distances between F1 and F7, shared by most of the oxazolines, reveal useful information for the further development of novel ligands (Figure 5). 

Indeed, for optimal PM mapping, the two hydrophobic rings should be folded towards each other, as exemplified by F1: Aro|Hyd and F2:Hyd, within a distance of 3.99 A. While F2:Hyd should be in the proximity of the H-bonding groups (2.09 A and 3.42 A, respectively) from the main F6:Acc and F7:Don moieties, the terminal aromatic ring F1:Aro|Hyd should be maintained at ideal distances of 4.40 A and 6.32 A from the aforementioned F6:Acc and F7: Don. 

On the other hand, the introduction of other H-bonding groups—as exemplified by the F3:Don2 F4:Acc2, F5:Don2 features placed at 8.48 A, 5.35 A and 7.33 A from F1:Aro|Hyd—are well-tolerated, representing the final cut-off values for the rational design of new analogues. 

**Figure 6:**
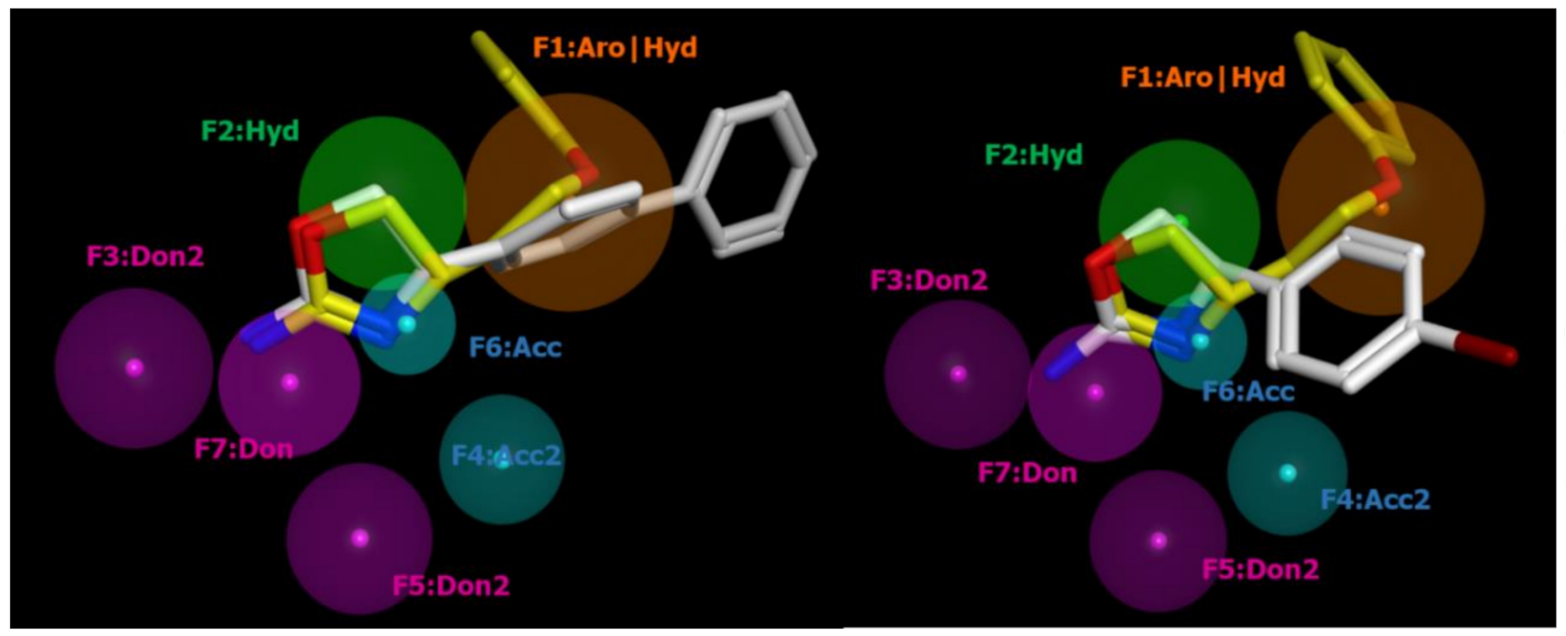
Pharmacophore features shared by most of the oxazolines acting as *h*TAAR1 agonists. The most potent derivative, **20b**, is depicted by bold sticks (colored as in Figure 4). The less potent analogues, **30b** (**left**) and **27b** (**right**), are also shown for comparison (*C* atoms colored in white).

Compound **20b** (pEC_50_ = 8.05)—chosen as representative oxazoline—fulfills these requirements, through the primary amine group, the oxazoline ring and the terminal phenyl ring, which proved to be properly folded thanks to the presence of the ethoxy chain (Figure 6). 

Conversely, the less potent analogues **30b** (pEC_50_ = 5.57) and **27b** (pEC_50_ = 5.00), used as external derivatives, were too bulky compared to **20b** or were folded differently with respect to the previously discussed pharmacophore model (Figure 6). As a consequence, they experienced lower potency values as *h*TAAR1 agonists. 

**Figure 7:**
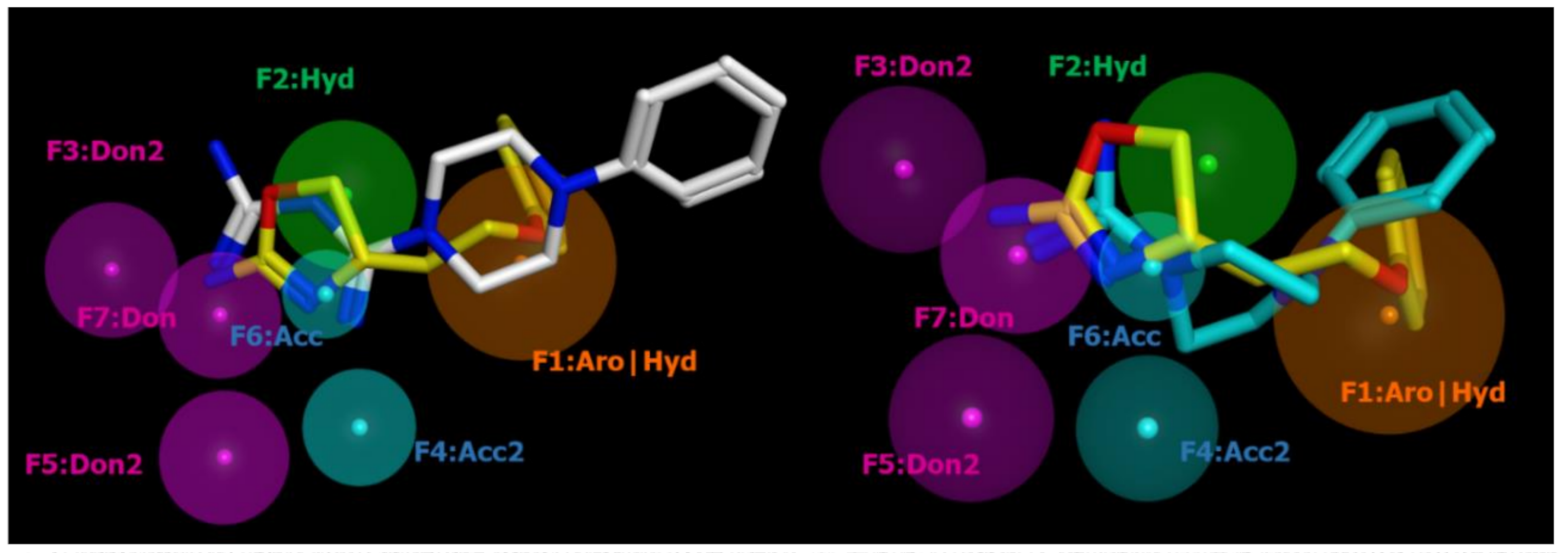
Pharmacophore features shared by most of the oxazolines acting as *h*TAAR1 agonists (Table S1). The most potent derivative **20b** is depicted by bold sticks (colors as in Figure 4). The PM mapping of the in-house developed *h*TAAR1 agonist **1a** (**left**) and the newly designed and synthesized prototype **1** (**right**) are also shown for comparison (*C* atoms colored in white and cyan, respectively).

On this basis, we decided that it would be interesting to compare the previously identified 1-phenyl-4-biguanylpiperazine **1a** of SET2 (Figure 2) and compound **1*,*** as the prototype of the newly developed ligands (**1**–**16**), with the structural information derived from the oxazoline analysis. As shown in Figure 7 (left), the biguanide moiety of **1a** was placed in the proximity of the amino-oxazoline portion, showing additional H-bonding features to those described by the pharmacophore modeling, while the phenylpiperazine group was too flat compared with the phenoxy ethyl chain of **20b**. This **1a** positioning quite closely resembles that previously described for oxazoline **30b**. Accordingly, **1a** (pEC_50_ = 5.86) and **30b** (pEC_50_ = 5.57) experienced comparable potency ability as *h*TAAR1 agonists.

On the other hand, the amidine moiety of **1** highly mimics the polarity trend of the oxazoline ring as well as of the primary amine group of **20b**, while the piperazine spacer allowed the compound to be folded in the proximity of the **20b** phenoxy group (Figure 7, right).

**Figure 8:**
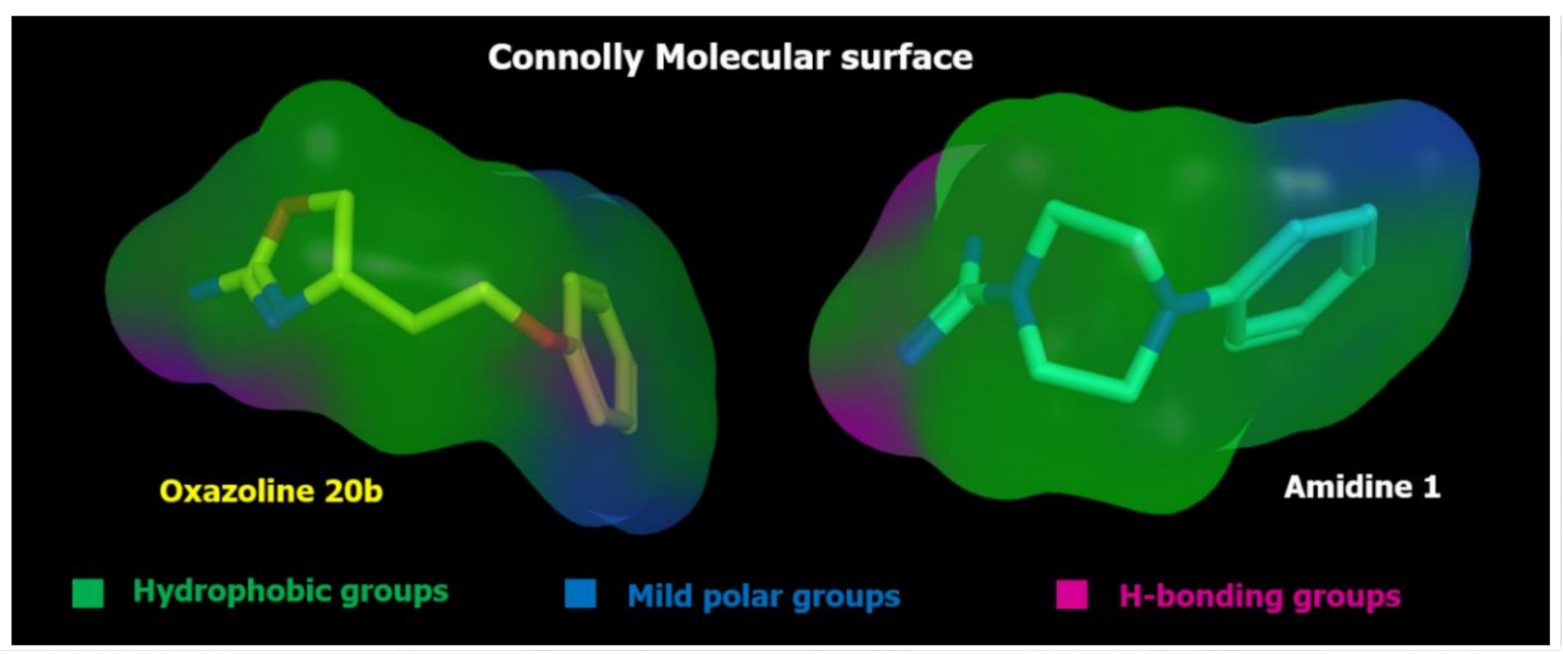
Comparison of the Connolly molecular surface properties of **20b** (**left**) and **1** (**right**) in terms of hydrophobic, polar and H-bonding features. Agonists are shown as bold sticks (*C* atoms colored in yellow and white, respectively).

Calculations of polar and hydrophobic properties at the molecular surface of **1** and **20b** revealed similar features in terms of H-bonding groups and lipophilic substituents, supporting the idea of modifying the biguanide–piperazine substituent with the amidine-containing group (Figure 8). 

### 2.3. Chemistry

Compounds **1** [32], **8** [33], **14** [34] were already described in the literature as free bases, while **2** [35], **4** [35,36], **5** [36] and **13** [35] were reported as hemisulfates and **10**, **11** [37] as benzoate salts. Since our synthetic procedure allowed for the yield of the title compounds as pure monohydrochlorides, their experimental properties have been reported herein.

**Scheme 1:**
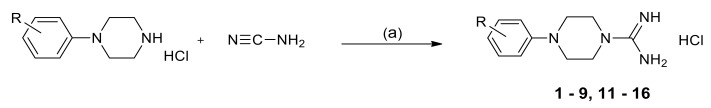
Reagents and conditions: (a) fusion at 220 °C, 30 min.

**Scheme 2:**
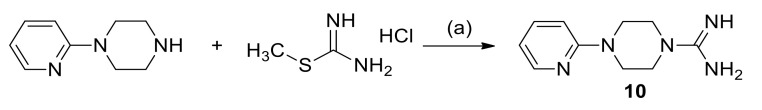
Reagents and conditions: (a) H_2_O, reflux 2h.

An equimolar mixture of the proper 1-aryl(heteroaryl)piperazine monohydrochloride and cyanamide was fused at 220 °C for 30 min, leading to 1-amidino-4-arylpiperazines of Scheme 1. The reaction failed, starting from the 1-(pyridin-2-yl)piperazine; thus, compound **10** was prepared by refluxing an aqueous solution of the piperazine derivative with S-methylisothiourea for 2 h with stirring (Scheme 2).

### 2.4. Biological Studies and SAR

Compounds **1**–**16** were evaluated for functional activity at *h*TAAR1 and for cytotoxicity against the Vero 76 cell line (Table 2). The activity of the compounds was measured using human embryonic kidney 293 (HEK-293T) cells co-transfected with plasmids encoding *h*TAAR1 and a cAMP Bioluminescence Resonance Energy Transfer (BRET) biosensor, or empty vector as a control. TYR was used as a positive control for agonism. Firstly, all the compounds were tested at 10 μM either for agonistic or antagonistic activity. Then, for the active compounds, a dose–response experiment was performed using concentrations in the range from 10 nM to 10 μM in order to calculate their corresponding EC_50_ values. The E_max_ value for the functional activity data defines the degree of functional activity compared to 100% for a full agonist TYR. The compounds sharing an E_max_ > 85% at *h*TAAR1 were considered as full agonists.

The new truncated compounds were synthesized from the piperazino-based biguanide prototypes of SET2 by replacing the more rigid biguanide chain with the amidino moiety. Most of them showed a potent agonism activity at *h*TAAR1, reaching the nanomolar potency (EC_50_ = 20–370 nM) of the endogenous ligands and of the above-reported RO compounds.

As observed for the previously developed biguanide series (SET1 and SET2), the lipophilic Cl and CH_3_ groups on the aromatic ring were confirmed as being valuable substitutions that were able to enhance the activity at *h*TAAR1 (**2**–**4**, **15**, **16**); the OCH_3_ substituent also proved to be effective (**6**, **7**). In this new series, the main determinant for the TAAR1 agonism ability seemed to be the result of an appropriate combination between the steric hindrance and position of the substituent on the phenyl ring rather than the different nature of substituents, since both electron-withdrawing and electron donor groups were tolerated. Regarding each series of isomers (**3**–**5** and **6**–**8**), the *ortho*-position (**3**, **6**) was more efficient than the *meta* ones (**4**, **7**), for which a lower level of activity was observed, while the *para*-substitution provided a negative outcome (**5**, **8**). Interestingly, the same comparable trend was shown by the disubstituted compounds, **15** and **16**, where the 2,3-diCl substitution led to a 3,5-fold increase in TAAR1 activity (EC_50_ = 20 nM) with respect to the 3,4-diCl one (EC_50_ = 71 nM). As a result, the *ortho*-substitution seems to impose a more appropriate spatial geometry to the molecule that entails the most relevant TAAR1 agonism profile, as experienced, in the following order, by compounds **15**, **2**, **6** and **3**.

From the analysis of the steric size of the different substituents on the phenyl ring, the presence of the larger 2-CH_3_ and 2-OCH_3_ groups is seen to correlate with a larger gain in the potency profile (**2** and **6**, EC_50_ = 30 and 93 nM, respectively) than the corresponding smaller 2-Cl (**3**, EC_50_ = 160 nM) and 2-F (**13**, inactive) ones, with the only exception being 2-CN (**12**, inactive). The same trend was also observed for the *meta*-substitution, as the activity decreased, passing from CF_3_ (**9**, EC_50_ = 64 nM) and OCH_3_ (**7**, EC_50_ = 244 nM) groups to the smaller Cl atom (**4**, EC_50_ > 2000 nM). Another permitted substitution was represented by the 2-pyridine ring (**10**) in place of the phenyl ring, whose efficacy nevertheless decreased by some orders of magnitude in the low micromolar range (EC_50_ > 2000 nM). 

**Figure 9:**
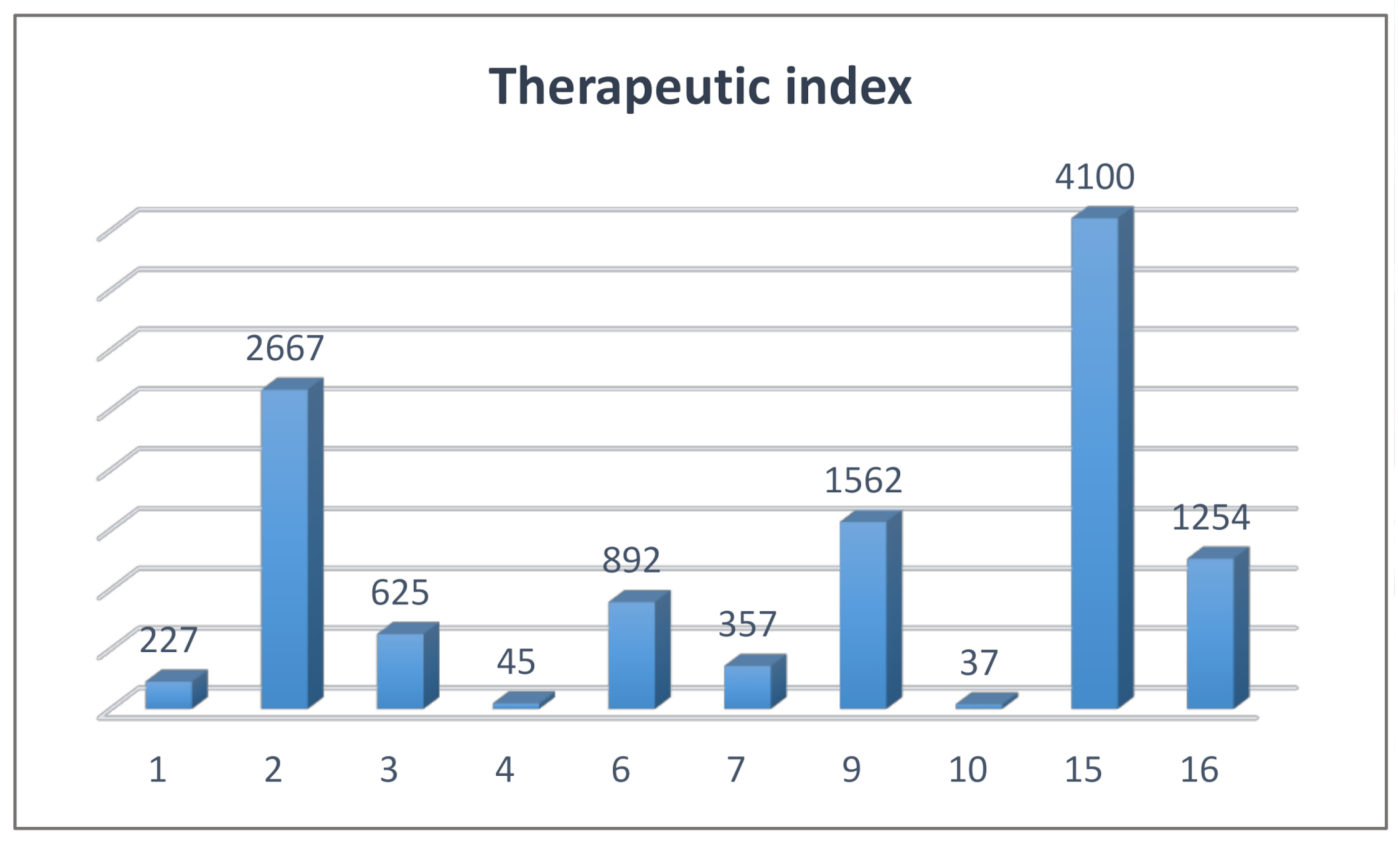
Therapeutic index (CC_50_/EC_50_) values of the active 1-amidino-4-arylpiperazines.

Importantly, this class of compounds elicited additional interest for its general low toxicity (CC_50_ > 70 µM, Table 1) against Vero-76 cells; accordingly, the corresponding therapeutic index (defined as the ratio of CC_50_ to EC_50,_ Figure 9) ranged from 625 (**3**) to 4100 (**15**) for the most active compounds (**15**, **2**, **9**, **16** and **6**), pointing to a very good safety profile.

On this basis, the molecular simplification of the biguanide skeleton of piperazino-based biguanides (SET2) has proven to be a valid strategy, which allowed for the identification of the optimal features for an efficient TAAR1 agonism behavior with the amidino group as basic motif and a planar aromatic ring as the lipophilic substituent, properly spaced by the bifunctional piperazine ring. Therefore, our results provide the foundation to further investigate these 1-amidino-4-arylpiperazine derivatives in order to assess their TAAR1 selectivity profile and make them worthy of structural optimization towards improved derivatives endowed with well-suited drug-like properties. Indeed, previous in vivo and in vitro studies of amidino piperazines described the ability of some compounds (corresponding to the present derivatives **1**, **4** and **5**) to produce a sympathomimetic effect, causing a rise in the blood pressure, potentiating the pressor effect induced by tyramine and norepinephrine (NE). These effects were correlated to the inhibition of monoamine oxidase (MAO) activity and of the restoration of NE in its storage site as a preliminary point, even if the authors did not exclude the possibility of other unknown mechanisms [36]. Furthermore, the intraperitoneal administration of 100 mg/kg of 1-phenyl-4-guanylpiperazine (**1**) increased the concentration of NE by about 34.5% in the brain and 42.7% in the heart of rats [38]. These findings pointed out an appropriate bioavailability profile for this class of compounds, which were demonstrated to interfere with the adrenergic system, both in the brain and in the periphery, a finding that was also observed for other classes of TAAR1 ligands thus far reported.

### 2.5. Docking Studies

As the last step in this work, we performed molecular docking studies of the newly developed amidine-containing derivatives exploiting our *h*TAAR1 model [29,31]. Specifically, we focused on the most promising derivatives, **1**, **2**, **6** and **15**, and on β-PEA as the reference compound. Additional docking studies of the previously cited oxazolines **20b**, **27b** and **30b** allowed us to validate the whole computational study and to better delineate the SAR profile featured by the new series of TAAR1 agonists (see scoring functions in Table S2). 

**Figure 10:**
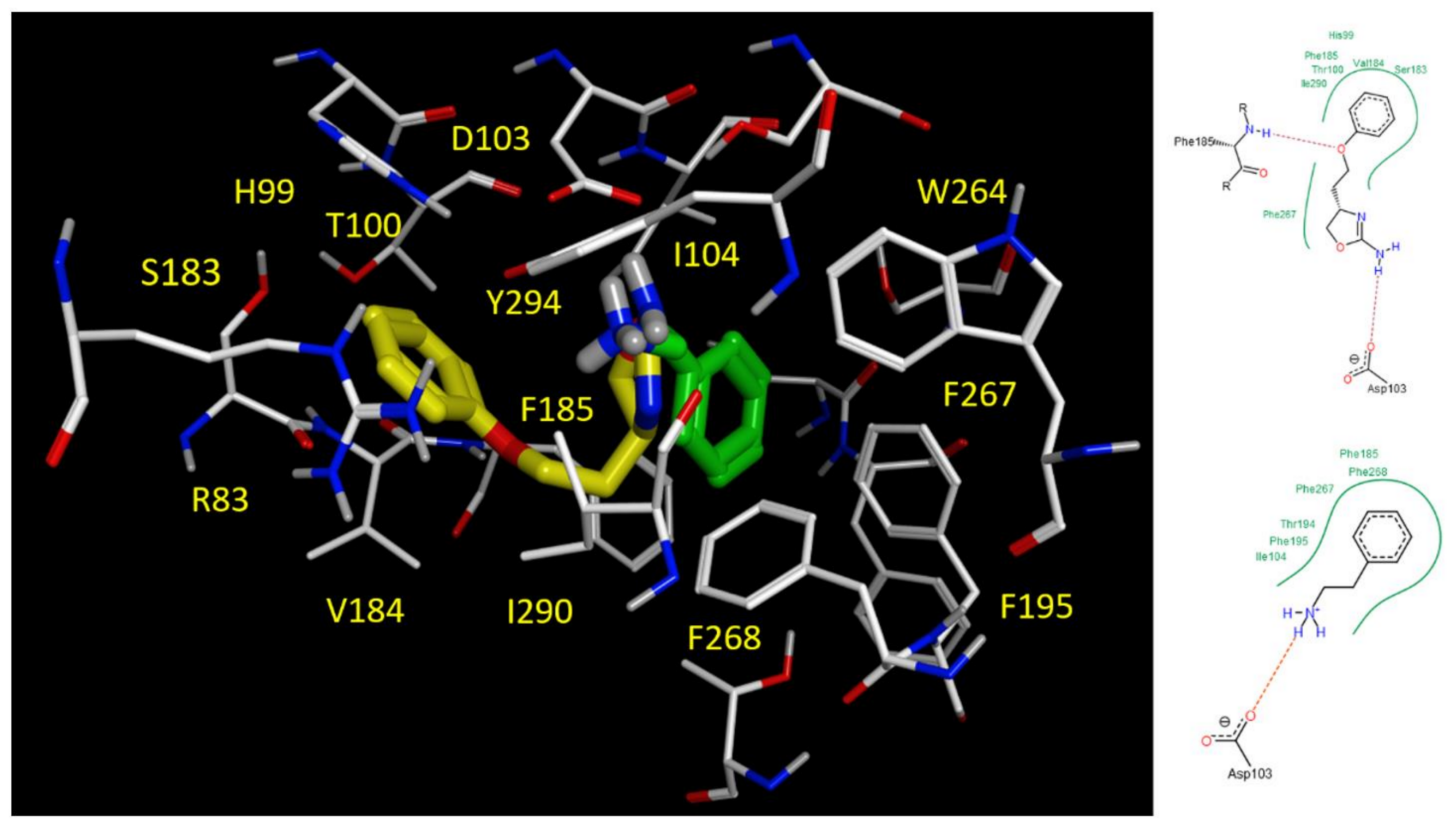
Docking mode of the potent oxazoline **20b** (*C* atom; yellow) and of β-phenylethylamine (β-PEA) (*C* atom; green) at the *h*TAAR1 putative binding site (**left**). Ligands are shown as sticks. The most relevant residues are labelled. A schematic perspective of the most relevant H-bonds experienced by the two agonists is also shown (**right**).

In particular, the docking mode of the endogenous TAAR1 agonist β-PEA (pEC_50_ = 6.70 M) revealed one ionic interaction between the protonated nitrogen atom and D103 while the aromatic ring of the compound was stabilized at the receptor crevice by π–π stacking and Van der Waals contacts with F185, F195, F267 and F268 (Figure 10). 

Maintaining a flexible chain connecting a basic core to a terminal aromatic ring, as experienced by the potent agonist **20b** (pEC_50_ = 8.05 M), allowed the compound to be properly folded within the GPCR cavity, in order to display a further H-bond between the ethoxy alkyl chain and the F185 backbone (Figure 10). On the other hand, the primary amine group linked to the oxazoline ring of **20b** was H-bonded to the D103 residue while the amino-oxazoline group and the phenyl ring were engaged in polar contacts and cation–π interaction with Y294 and H99, respectively. As a consequence, **20b** revealed a high number of polar contacts within the protein binding site if compared to the endogenous ligand β-PEA, exhibiting higher potency values than this trace amine, featuring the necessary π–cation and H-bond interactions [29,31]. 

Contextually, the alkyl chain and the terminal phenyl ring are engaged in van der Waals contacts and π–π stacking with the side chains of V184, I290 and F185, respectively. A different point of view for the discussed docking poses is reported in Figure S1.

**Figure 11:**
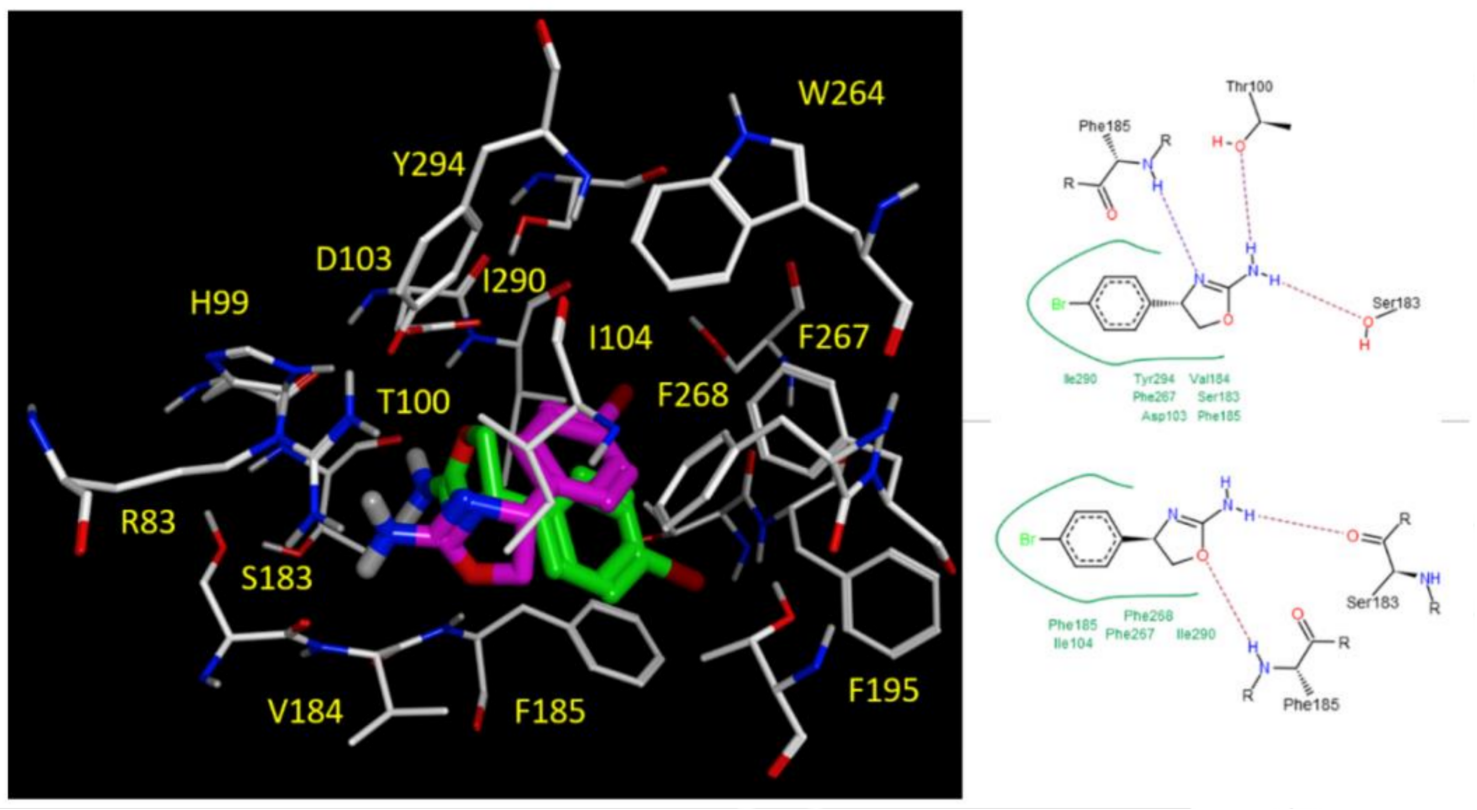
Docking positioning of the oxazolines **26b** (*C* atom; green) and **27b** (*C* atom; magenta) at the *h*TAAR1 putative binding site (**left**). Ligands are shown as sticks. The most relevant residues are labelled. A schematic perspective of the most relevant H-bonds experienced by the two agonists is also shown (**right**).

On the whole, the related scoring value obtained for this protein–agonist complex is in agreement with the potency observed for **20b** (pEC_50_ = 8.05 M, S = −99.1078) and supports the higher effectiveness of this compound compared with the endogenous ligand β-PEA (pEC_50_ = 6.70 M, S = −95.8775). In fact, the docking mode of β-PEA features the required key contact with D103 via the protonated nitrogen atom, along with hydrophobic interactions and π–π stacking between the ethyl chain and I290, and the phenyl ring with F195, F267 and F268, respectively. These findings are validated by the docking positioning derived for the **20b** rigid analogues **26b** (pEC_50_ = 6.82 M), **27b** (pEC_50_ = 5.00 M) and **30b** (pEC_50_ = 5.57 M), supporting a relevant role played by interacting with D103 and F185 to stabilize the affective agonist at the receptor binding site. Concerning the two enantiomers **26b** (*S*) and **27b** (*R*), only the first one displayed promising potency values as a hTAAR1 agonist thanks to polar contacts and H-bonds between the primary amine group and D103 and T100, S183, respectively (Figure 11). This kind of positioning allowed the compound to exhibit π–π stacking and Van der Waals contacts with F185, F267 and V184, F85 and I290.

The modest agonist **27b** (*R*) experienced a shifted docking mode compared to **26b** (*S*), moving the oxazoline ring far from the D103 residue in order to detect only two H-bonds with T100 and F185 (Figure 11) and therefore motivating the lower potency trend of this derivative. However, **27b** was able to maintain a number of hydrophobic and π–π contacts with the surrounding residues F185, F267 and F268, supporting the idea of a main aromatic core tethered to proper H-bonding features.

We reasoned that that the limited flexibility and dimensions of **27b,** especially when combined with a small hydrophobic substituent at the *para* position of the terminal phenyl ring, could impair the capacity of the compound to properly occupy the receptor crevice with the expense of polar contacts with D103, due to a consistent number of hydrophobic interactions with the GPCR cavity. Interestingly, these results are also in accordance with the SAR developed within the amidine series, with **5**, **8**, **14** and **16** being inactive as *h*TAAR1 agonists. A bulkier substituent may be preferred to arrange and better stabilize the ligand at the receptor binding site.

Accordingly, the oxazoline **30b** (pEC_50_ = 5.57; S = −84.7643), bearing a biaryl substituent connected to the oxazoline core, displayed several van der Waals contacts and aromatic stacking with I104, V184 and with F185, W264, F267, respectively, while the amino-oxazoline ring was H-bonded to T100, S183 and F185 (see Figure S2). In particular, this positioning allowed the compound to exhibit higher potency than **27b**, as confirmed by the calculated scoring functions.

**Figure 12:**
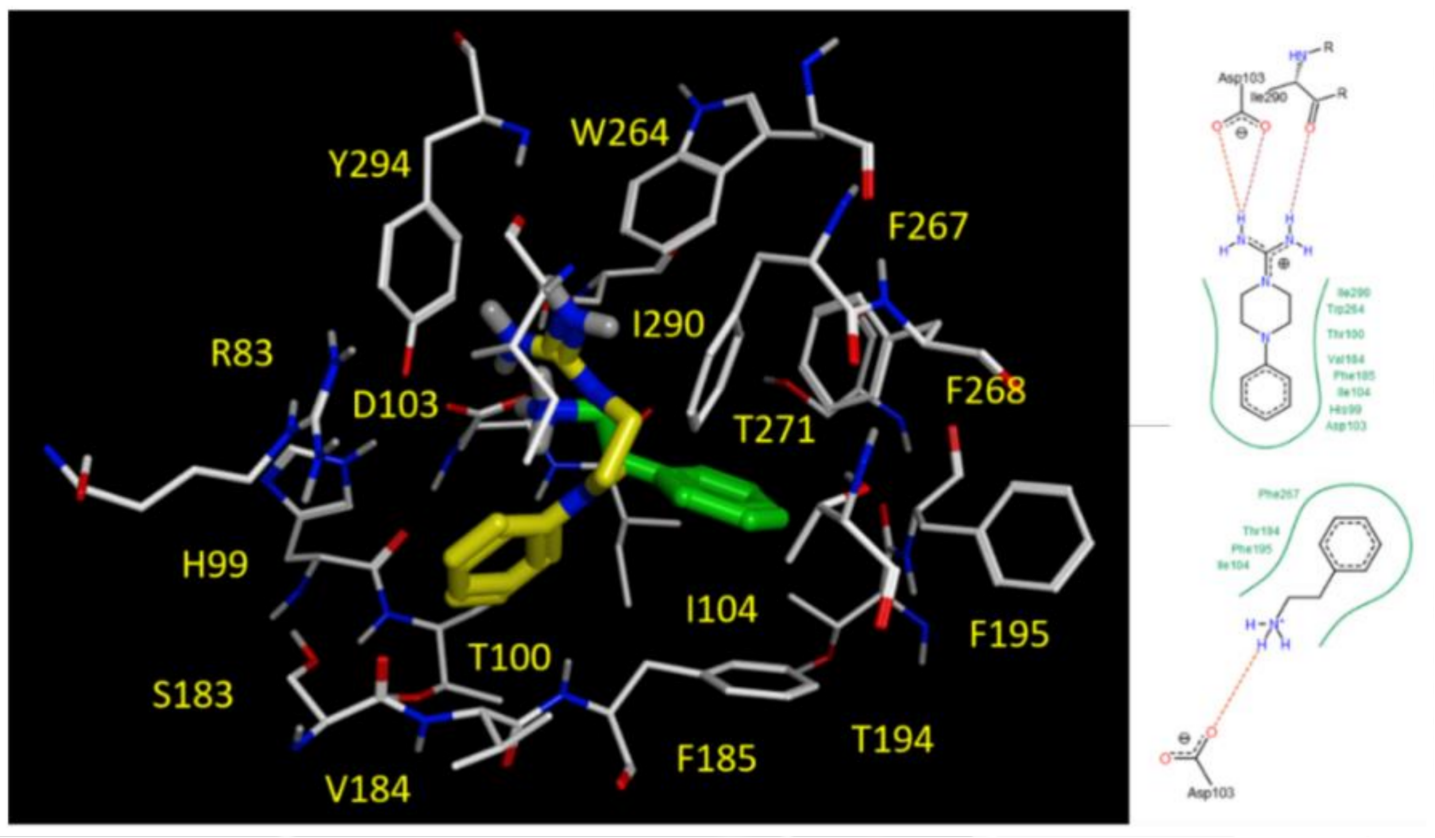
Docking positioning of the amidine-containing derivatives **1** (*C* atom; yellow) and β-PEA (*C* atom; green) as *h*TAAR1 agonists at the receptor crevice (**left**). Ligands are shown as sticks. The most relevant residues are labelled. A schematic perspective of the most relevant H-bonds experienced by the two agonists is also shown (**right**).

**Figure 13:**
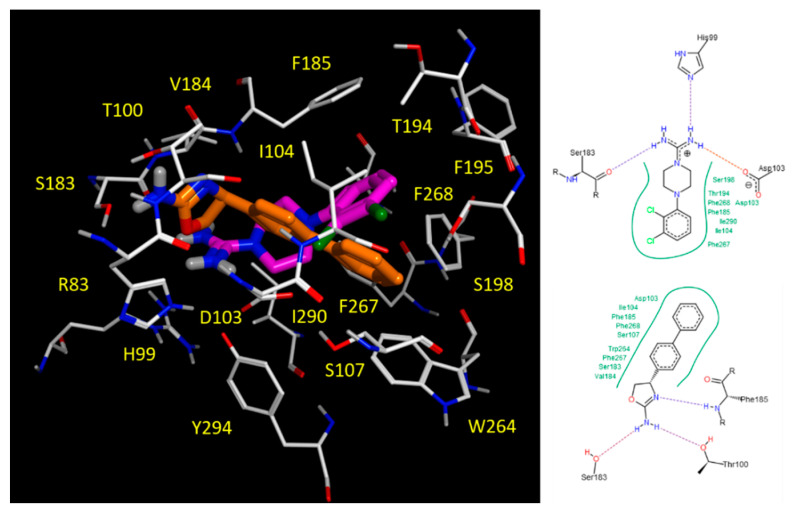
Docking positioning of the amidine-containing derivatives **15** (*C* atom; magenta) and of the oxazoline **30b** (*C* atom; orange) as *h*TAAR1 agonists at the receptor crevice (**left**). Ligands are shown as sticks. The most relevant residues are labelled. A schematic perspective of the most relevant H-bonds experienced by the two agonists is also shown (**right**).

Concerning the newly developed amidine-containing derivatives, both the prototype **1** and the most promising derivative **15** slightly shift their position within the protein cavity, bringing the amidine moiety into the proximity of the receptor residues H99 and D103, thereby engaging their side chains in H-bond contacts (Figure 12 and Figure 13). Moreover, the folded piperazine, in tandem with the presence of substituents at the phenyl ring, guides the aromatic core and the amidine moiety towards the hydrophobic cavity delimited by I104, V184, F185, W264, I290.

As shown in Figure 12, the unsubstituted compound **1** (pEC_50_ = 6.43 M, S = −85.7222) orients the aromatic core towards H99 and V184, thereby featuring van der Waals contacts and cation–π interactions, while the piperazine ring is involved in Van der Waals contacts with F267. 

Moreover, the amidine moiety was H-bonded to D103 and I290, therefore being stabilized within the receptor cavity. Notably, the discussed docking mode of compound **1** was quite comparable with that of β-PEA, with the exception of π–π stacking with F267 and F268, due to the rigid piperazine in place of the flexible ethyl chain (Figure 12). These findings are in agreement with the higher pEC_50_ values of the endogenous trace amine β-PEA (pEC_50_ = 6.70 M) in comparison with that of the amidine derivative **1**. A different point of view for the discussed docking poses is reported in Figure S3.

Regarding the most potent analogue **15** (pEC_50_ = 7.70 M, S = −95.0332), the reported docking mode revealed the effective positioning of the agonist within the larger cavity of the hTAAR1 receptor, including F185, S198, W264, F267 and F268, featuring π–π stacking and Van der Waals contacts thanks to the aryl moiety. This kind of docking mode allows the compound to better match the binding site features, exhibiting a higher number of polar contacts with H99, D103 and S183 via its amidine moiety (Figure 13).

This is in accordance with the higher effectiveness of **15** (pEC_50_ = 7.70 M) compared with the oxazoline **30b** (pEC_50_ = 5.57), as shown in Figure 13. Indeed, the amidine moiety is a good bioisostere of the amino-oxazoline ring while the 2,4-dichlorophenyl piperazine portion proved to be better substituent than the biaryl moiety to efficiently bind the GPCR crevice. The positioning of compound **15** allowed the agonist to detect stronger polar and H-bond contacts with D103 and S183 than **30b**. 

**Figure 14:**
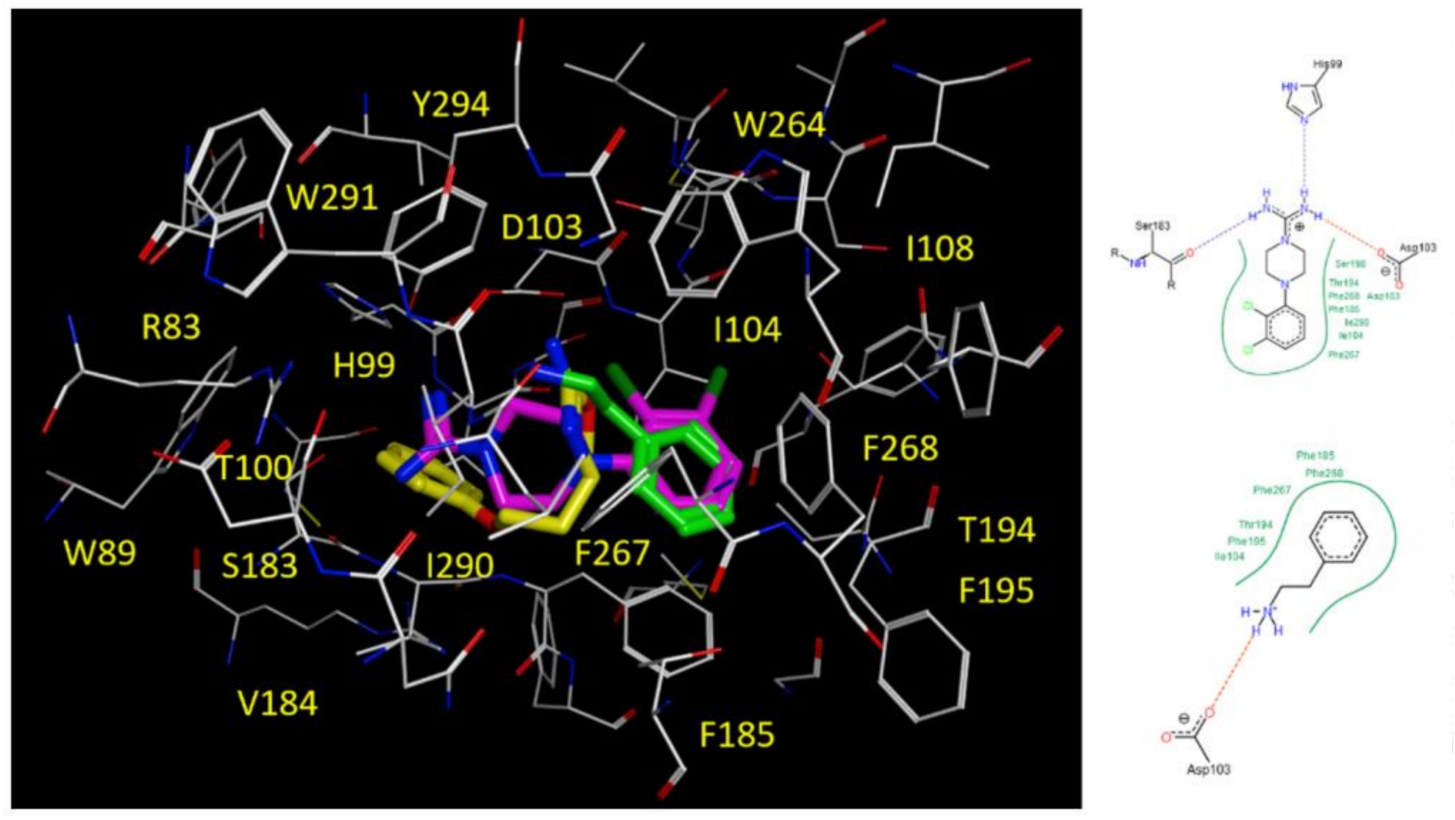
Docking positioning of potent oxazoline **20b** (*C* atom; yellow), of the amidine-containing derivative **15** (*C* atom; magenta) and of the endogenous agonist β-PEA (*C* atom; green) as *h*TAAR1-targeting compounds (**left**). Ligands are shown as sticks. The most relevant residues are labelled. A schematic perspective of the most relevant H-bonds experienced by **15** and β-PEA is also shown (**right**).

A good similarity in terms of putative bioactive conformation can be noticed by comparing the docking mode of compound **15** with that previously discussed for **20b** (pEC_50_ = 8.05 M) and β-PEA (pEC_50_ = 6.70 M), as shown in Figure 14.

While the 2,4-dichlorophenyl group of **15** highly mimicked the aromatic ring of β-PEA, the amidine portion displayed additional H-bonds than the trace amine, supporting the higher potency of **15** in comparison to β-PEA. On the other hand, the amidine moiety guaranteed the required polar interactions with H99 and D103, as featured by **20b** and additional contacts with S183, while the piperazine spacer overlapped the ethoxyamine chain of the oxazoline, lacking H-bonds with F185. As a result, **15** experienced quite comparable potency with respect to **20b**.

Finally, the introduction of an electron donor substituent at the *ortho* position of the phenyl ring led to the 2-methyl- or 2-methoxy-phenyl substituted analogues **2** and **6** exhibiting adequate *h*TAAR1 agonist ability. Both compounds show a docking mode comparable to that of **15**, with derivative **2** (pEC_50_ = 7.52 M, S = −94.6754) being more effective than **6** (pEC_50_ = 7.03 M, S = −94.5612). Indeed, the 2-methylphenyl ring is better placed within the receptor cavity, mimicking the role played by the dichloro-substituted phenyl ring of **15**. In any case, the amidine moiety of **2** and **6** properly displays the key contacts with H99 and D103 (see Figure S4).

### 2.6. Prediction of ADMET Properties

Nowadays, applying in silico methods for the computational prediction of descriptors related to the pharmacokinetic (PK) and toxicity profile of novel molecules represents a useful tool that is accelerating the lead compound discovery process [39]. A number of parameters involved in the absorption, distribution, metabolism, excretion and toxicity properties (ADMET) of putative druglike derivatives can be managed in silico, and we successfully applied this computational approach on our first series of TAAR1 agonists [29].

Thus, herein, we explored the putative ADMET profile of the newly developed TAAR1 agonists **1**–**16** as well as of the endogenous ligand β-PEA and of the oxazoline derivative RO5263397, taken as reference drug-like derivatives. In particular, the favorable pharmacokinetic profile of RO5263397 has been experimentally determined [18]. Briefly, RO5263397 was characterized by an acceptable human hepatocyte clearance and the inhibitory activity of the cytochrome isoforms, exhibiting in vitro safety profile performed by a (negative) AMES/MNT test and hERG IC_50_ evaluation. 

In this work, for the aforementioned amidine-based derivatives, the logarithmic ratio of the octanol–water partitioning coefficient (cLogP), the ability to pass the blood–brain barrier (BBB) to the extent of BBB permeation (LogBB), the rate of passive diffusion permeability (LogPS), human intestinal absorption (HIA), the volume of distribution (Vd), the role played by plasmatic protein binding (%PPB), and the ligand affinity toward human serum albumin (LogKa ^HSA^) were all taken into account in order to determine the putative value of the oral bioavailability as a percentage (%F) (see Table 3).

The putative metabolism and toxicity profiles of any compound were determined on the basis of a number of descriptors such as the ability to interact with the endocrine system and to inhibit the hERG channel, as well as to act as cytochrome P450 3A4 and 2D6 inhibitors or substrates (see Table 4).

As shown in Table 3, all the newly synthesized compounds with the exception of compound **11** were predicted as being able to pass through the BBB thanks to organic cation transporters. Among them, the promising compounds **15** and **16** were endowed with comparable lipophilicity values with respect to the reference compounds β-PEA and RO5263397, turning in favorable values of human intestinal absorption (HIA = 92–95%). While the reported prediction of volume of distribution (Vd) for **15** (Vd = 1.5 L/Kg; %PPB = 50.18%) and **16** (Vd = 1.6 L/Kg; %PPB = 50.85%) was noticeably lower than that of the two references (Vd = 2.4–3.0 L/Kg; %PPB = 27.57–34.79%), the potential binding to the plasmatic proteins fell in the allowed ranges (spanning from 40% to 80%), being higher than those of β-PEA and RO5263397. Accordingly, the predicted LogKa ^HAS^ values were quite comparable (compare **15**, **16** (LogKa ^HSA^ = 3.52–3.57) to β-PEA and RO5263397 (LogKa ^HSA^ = 2.78–3.12)). Regarding **2**, **6** and **9**, only the last one was endowed with a proper PK profile based on the calculated cLogP (**9** cLogP = 1.26), HIA% (>70%), %PPB (>50%) and %F (>50%), as reported in Table 3.

According to an overall analysis of the descriptors listed in Table 4, none of the compounds proposed here should be involved in genotoxicity events such as binding with the endocrine system, while only amidine **9** is predicted as a putative hERG inhibitor, being accompanied by a high reliability index R.I = 0.56. Furthermore, none of them were proven to inhibit the cytochrome P450 3A4, as only the reference agonist RO5263397 was the substrate for this enzyme. On the other hand, the endogenous amine β-PEA as well as the synthesized TAAR1 agonists **1**–**16** were predicted as substrates for CYP2D6 with high reliability indexes (RIs) (>50%).

## 3. Materials and Methods 

### 3.1. Chemistry

#### 3.1.1. General Information

Chemicals, solvents and reagents (R-phenylpiperazines) used for the syntheses were purchased from Sigma-Aldrich (Milan, Italy), and were used without any further purification. Melting points (uncorrected) were determined with a Büchi apparatus (Milan, Italy). ^1^H NMR spectra and ^13^C NMR spectra were recorded on a Varian Gemini-200 instrument at 200 and 50 MHz, respectively; DMSO-*d*_6_; δ in ppm rel. to Me_4_Si as internal standard. J in Hz. Elemental analyses were performed on a Flash 2000 CHNS (Thermo Scientific, Milan Italy) instrument in the Microanalysis Laboratory of the Department of Pharmacy, University of Genova. The results of elemental analyses indicated that the purity of all compounds was ≥95%.

#### 3.1.2. General Method for the Synthesis of 1-Amidino-4-Arylpiperazine Derivatives

A mixture of the proper phenylpiperazine hydrochloride (2.5 mmol) and cyanamide (2.5 mmol) was fused at 220 °C for 30 min. At r.t., the hard solid was rinsed with a mixture of EtOAc and Et_2_O and washed with the same solvents, affording the final products in the form of monohydrochlorides. For some highly hygroscopic monohydrochloride salts, melting points have not been reported, since they melted with decomposition in a wide range.

*4-Phenylpiperazine-1-carboximidamide hydrochloride* (**1**): Yield: 90%. M.p. 214–217 °C. ^1^H NMR (200 MHz, DMSO-*d*_6_): δ 7.76 (s, NH_2_^+^ exchange with D_2_O), 7.20 (pseudo s, 2ArH), 7.17–6.84 (m, 5H, 3ArH and NH_2_ exchange with D_2_O), 3.72–3.51 (m, 4H, 2CH_2_ piperazine), 3.21–3.08 (m, 4H, 2CH_2_ piperazine). ^13^C NMR (50 MHz, DMSO-*d*_6_): δ 161.1, 149.7, 128.7 (2C), 122.6, 117.0 (2C), 54.3 (2C), 48.1 (2C). Anal. Calcd. For C_11_H_16_N_4_·HCl: % C, 54.88; H, 7.12; N, 23.27. Found: C, 54.63; H, 6.92; N, 23.68.

*4-(o-Tolyl)piperazine-1-carboximidamide hydrochloride* (**2**): Yield: 83%. M.p. 159–162 °C. ^1^H NMR (200 MHz, DMSO-*d*_6_): δ 7.80 (s, NH_2_^+^ exchange with D_2_O), 7.43 (pseudo s, 1ArH), 7.23–6.86 (m, 5H, 3ArH and NH_2_ exchange with D_2_O), 3.62–3.56 (m, 4H, 2CH_2_ piperazine), 3.27–2.97 (m, 4H, 2CH_2_ piperazine), 2.41 (s, CH_3_). ^13^C NMR (50 MHz, DMSO-*d*_6_): δ 162.3, 149.9, 131.1, 129.8, 126.7, 122.2, 117.9, 52.4 (2C), 47.1 (2C), 19.6. Anal. Calcd for C_12_H_18_N_4_·HCl: % C, 56.57; H, 7.52; N, 21.99. Found: C, 56.64; H, 7.80; N, 21.69.

*4-(2-Chlorophenyl)piperazine-1-carboximidamide hydrochloride* (**3**): Yield: 67%. ^1^H NMR (200 MHz, DMSO-*d*_6_): δ 7.79 (s, NH_2_^+^ exchange with D_2_O), 7.35–7.32 (m, 1ArH), 7.19–6.97 (m, 5H, 3ArH and NH_2_ exchange with D_2_O), 3.71–3.54 (m, 4H, 2CH_2_ piperazine), 3.17–2.91 (m, 4H, 2CH_2_ piperazine). ^13^C NMR (50 MHz, DMSO-*d*_6_): δ 162.1, 151.1, 130.5, 127.3, 126.2, 123.0, 120.1, 51.7 (2C), 46.3 (2C). Anal. Calcd for C_11_H_15_ClN_4_·HCl: % C, 48.01; H, 5.86; N, 20.36. Found: C, 48.11; H, 6.04; N, 20.29.

*4-(3-Chlorophenyl)piperazine-1-carboximidamide hydrochloride* (**4**): Yield: 45%. ^1^H NMR (200 MHz, DMSO-*d*_6_): δ 7.61 (s, NH_2_^+^ exchange with D_2_O), 7.42 (s, 1 ArH), 7.26–7.19 (m, 1ArH), 7.09–6.88 (m, 4H, 2ArH and NH_2_ exchange with D_2_O), 3.57 (pseudo s, 4H, 2CH_2_ piperazine), 3.21 (pseudo s, 4H, 2CH_2_ piperazine). ^13^C NMR (50 MHz, DMSO-*d*_6_): δ 161.4, 151.9, 139.1, 130.7, 120.5, 117.8, 113.5, 51.2 (2C), 46.7 (2C). Anal. Calcd for C_11_H_15_ClN_4_·HCl: % C, 48.01; H, 5.86; N, 20.36. Found: C, 48.14; H, 5.96; N, 20.04.

*4-(4-Chlorophenyl)piperazine-1-carboximidamide hydrochloride* (**5**): Yield: 52%. ^1^H NMR (200 MHz, DMSO-*d*_6_): δ 7.67 (s, NH_2_^+^ exchange with D_2_O), 7.14–6.89 (m, 6H, 4ArH and NH_2_ exchange with D_2_O), 3.58–3.49 (m, 4H, 2CH_2_ piperazine), 3.16–3.02 (m, 4H, 2CH_2_ piperazine). ^13^C NMR (50 MHz, DMSO-*d*_6_): δ 161.9, 150.6, 129.1 (2C), 123.7, 116.5 (2C), 49.6 (2C), 45.7 (2C). Anal. Calcd for C_11_H_15_ClN_4_·HCl: % C, 48.01; H, 5.86; N, 20.36. Found: C, 47.91 H, 5.96; N, 20.36.

*4-(2-Methoxyphenyl)piperazine-1-carboximidamide hydrochloride* (**6**): Yield: 90%. ^1^H NMR (200 MHz, DMSO-*d*_6_): δ 7.58 (s, NH_2_^+^ exchange with D_2_O), 7.12–6.84 (m, 6H, 4ArH and NH_2_ exchange with D_2_O), 3.72 (s, OCH_3_), 3.47 (pseudo s, 4H, 2CH_2_ piperazine), 3.23 (pseudo s, 4H, 2CH_2_ piperazine). ^13^C NMR (50 MHz, DMSO-*d*_6_): δ 162.7, 151.7, 142.5, 124.3, 122.6, 118.1, 113.7, 57.3, 50.7 (2C), 47.1 (2C). Anal. Calcd for C_12_H_18_N_4_O·HCl: % C, 53.23; H, 7.07; N, 20.69. Found: C, 53.46; H, 7.02; N, 20.96.

*4-(3-Methoxyphenyl)piperazine-1-carboximidamide hydrochloride* (**7**): Yield: 43%. ^1^H NMR (200 MHz, DMSO-*d*_6_): δ 7.64 (s, NH_2_^+^ exchange with D_2_O), 7.18–7.11 (m, 1ArH), 6.89–6.41 (m, 5H, 3ArH and NH_2_ exchange with D_2_O), 3.74 (s, OCH_3_), 3.53–3.49 (m, 4H, 2CH_2_ piperazine), 3.39–3.17 (m, 4H, 2CH_2_ piperazine). ^13^C NMR (50 MHz, DMSO-*d*_6_): δ 161.9, 159.8, 149.7, 131.2, 111.3, 107.6, 103.2, 56.9, 49.8 (2C), 47.3 (2C). Anal. Calcd for C_12_H_18_N_4_O·HCl: % C, 53.23; H, 7.07; N, 20.69. Found: C, 53.28; H, 7.22; N, 20.51.

*4-(4-Methoxyphenyl)piperazine-1-carboximidamide hydrochloride* (**8**): Yield: 54%. ^1^H NMR (200 MHz, DMSO-*d*_6_): δ 7.71 (s, NH_2_^+^ exchange with D_2_O), 7.17–6.92 (m, 6H, 4ArH and NH_2_ exchange with D_2_O), 3.78 (s, OCH_3_), 3.61 (pseudo s, 4H, 2CH_2_ piperazine), 3.22 (pseudo s, 4H, 2CH_2_ piperazine). ^13^C NMR (50 MHz, DMSO-*d*_6_): δ 162.4, 154.6, 143.8, 116.2 (2C), 114.8 (2C), 56.6, 50.1 (2C), 47.3 (2C). Anal. Calcd for C_12_H_18_N_4_O·HCl: % C, 53.23; H, 7.07; N, 20.69. Found: C, 53.43; H, 7.16; N, 20.90.

*4-[(3-Trifluoromethyl)phenyl]piperazine-1-carboximidamide hydrochloride* (**9**): Yield: 31%. M.p. 170–173 °C. ^1^H NMR (200 MHz, DMSO-*d*_6_): δ 7.76 (s, NH_2_^+^ exchange with D_2_O), 7.58–7.01 (m, 6H, 4ArH and NH_2_ exchange with D_2_O), 3.58 (pseudo s, 4H, 2CH_2_ piperazine), 3.27 (pseudo s, 4H, 2CH_2_ piperazine). ^13^C NMR (50 MHz, DMSO-*d*_6_): δ 160.3, 157.6, 131.2 (q, J_C-F_ = 22.4 Hz), 128.3 (d, J_C-F_ = 22.35 Hz), 124.6 (d, J_C-F_ = 271.25 Hz), 122.1 (d, J_C-F_ = 20.9 Hz), 121.3 (q, J_C-F_ = 37.5 Hz), 116.7 (q, J_C-F_ = 36.7 Hz), 50.1 (2C), 44.6 (2C). Anal. Calcd for C_12_H_15_F_3_N_4_·HCl: % C, 46.68; H, 5.22; N, 18.15. Found: C, 46.79; H, 5.44; N, 18.41.

*4-(Pyrimidin-2-yl)piperazine-1-carboximidamide hydrochloride* (**11**): Yield: 29%. ^1^H NMR (200 MHz, DMSO-*d*_6_): δ 7.97–7.36 (m, 4H, 2H pyrim. And NH_2_^+^ exchange with D_2_O), 7.31–6.64 (m, 3H, 1H pyrim. And NH_2_ exchange with D_2_O), 3.71 (pseudo s, 4H, 2CH_2_ piperazine), 3.59 (pseudo s, 4H, 2CH_2_ piperazine). ^13^C NMR (50 MHz, DMSO-*d*_6_): δ 161.4, 159.1, 157.6 (2C), 112.1, 46.8 (2C), 44.3 (2C). Anal. Calcd for C_9_H_14_N_6_·HCl: % C, 44.54; H, 6.23; N, 34.63. Found: C, 44.49; H, 6.34; N, 34.34.

*4-(2-Cyanophenyl)piperazine-1-carboximidamide hydrochloride* (**12**): Yield: 61%. M.p. 236–238 °C. ^1^H NMR (200 MHz, DMSO-*d*_6_): δ 7.98–7.52 (m, 6H, 2ArH, NH_2_ and 7.77, s, NH_2_^+^superimposed, exchange with D_2_O), 7.28–7.07 (m, 2ArH), 3.64 (pseudo s, 4H, 2CH_2_ piperazine), 3.23 (pseudo s, 4H, 2CH_2_ piperazine). ^13^C NMR (50 MHz, DMSO-*d*_6_): δ 162.2, 154.5, 136.7, 130.6, 122.1, 118.6, 117.4, 106.2, 51.1 (2C), 46.3 (2C). Anal. Calcd for C_12_H_15_N_5_·HCl: % C, 54.24; H, 6.07; N, 26.35. Found: C, 54.00; H, 6.24; N, 26.18.

*4-(2-Fluorophenyl)piperazine-1-carboximidamide hydrochloride* (**13**): Yield: 57%. M.p. 145–148 °C. ^1^H NMR (200 MHz, DMSO-*d*_6_): δ 7.80 (s, NH_2_^+^ exchange with D_2_O), 7.43–6.81 (m, 6H, 4ArH and NH_2_ exchange with D_2_O), 3.63 (pseudo s, 4H, 2CH_2_ piperazine), 3.07 (pseudo s, 4H, 2CH_2_ piperazine). ^13^C NMR (50 MHz, DMSO-*d*_6_): ^13^C NMR (50 MHz, DMSO-*d*_6_): δ 161.9, 159.1 (d, J_C-F_ = 243.1 Hz), 141.9 (d, J_C-F_ = 9.7 Hz), 128.1, 123.7 (d, J_C-F_ = 7.2 Hz), 120.0(d, J_C-F_ = 6.3 Hz), 116.4 (d, J_C-F_ = 22.1 Hz), 59.3 (2C), 47.8 (2C). Anal. Calcd for C_11_H_15_FN_4_·HCl: % C, 51.07; H, 6.23; N, 21.66. Found: C, 50.84; H, 6.35; N, 21.28.

*4-(4-Fluorophenyl)piperazine-1-carboximidamide hydrochloride* (**14**): Yield: 53%. ^1^H NMR (200 MHz, DMSO-*d*_6_): δ 7.76 (s, NH_2_^+^ exchange with D_2_O), 7.41–6.84 (m, 6H, 4ArH and NH_2_ exchange with D_2_O), 3.61 (pseudo s, 4H, 2CH_2_ piperazine), 3.16 (pseudo s, 4H, 2CH_2_ piperazine). ^13^C NMR (50 MHz, DMSO-*d*_6_): δ 162.4, 157.8 (d, J_C-F_ = 244.5 Hz), 148.1, 120.2 (d, J_C-F_ = 7.6 Hz; 2C), 115.6 (d, J_C-F_ = 23.4 Hz; 2C), 52.3 (2C), 47.4 (2C). Anal calcd for C_11_H_15_FN_4_·HCl: % C, 51.07; H, 6.23; N, 21.66. Found: C, 50.90; H, 6.38; N, 21.43.

*4-(2,3-Dichlorophenyl)piperazine-1-carboximidamide hydrochloride* (**15**): Yield: 37%. ^1^H NMR (200 MHz, DMSO-*d*_6_): δ 7.86 (s, NH_2_^+^ exchange with D_2_O), 7.69–6.98 (m, 5H, 3ArH and NH_2_ exchange with D_2_O), 3.76–2.97 (m, 8H, 4CH_2_ piperazine). ^13^C NMR (50 MHz, DMSO-*d*_6_): δ 163.1, 149.2, 135.1, 128.2, 126.3, 125.8, 115.5, 51.9 (2C), 46.2 (2C). Anal. Calcd for C_11_H_14_Cl_2_N_4_·HCl: % C, 42.67; H, 4.88; N, 18.10. Found: C, 42.27; H, 4.94; N, 17.94.

*4-(3,4-Dichlorophenyl)piperazine-1-carboximidamide hydrochloride* (**16**): Yield: 45%. M.p. 235–237°C. ^1^H NMR (200 MHz, DMSO-*d*_6_): δ 7.53–7.38 (m, 3H, 1ArH and 7.45, s, N*H*_2_^+^superimposed, exchange with D_2_O), 7.19–7.15 (m, 1ArH), 7.03–6.91 (m, 3H, 1ArH and N*H_2_* exchange with D_2_O), 3.61 (pseudo s, 4H, 2C*H_2_* piperazine), 3.27 (pseudo s, 4H, 2C*H_2_* piperazine). ^13^C NMR (50 MHz, DMSO-*d*_6_): δ 162.6, 149.1, 131.9, 129.1, 120.2, 116.8, 114.7, 50.4 (2C), 46.7 (2C). Anal. Calcd for C_11_H_14_Cl_2_N_4_·HCl: % C, 42.67; H, 4.88; N, 18.10. Found: C, 42.59; H, 4.64; N, 18.29.

#### 3.1.3. Synthesis of 4-(Pyridin-2-yl)Piperazine-1-Carboximidamide Hydrochloride (10)

1-(Pyridin-2-yl)piperazine (2.65 mmol) was added to a solution of S-methylisothiourea hydrochloride (3.95 mmol) in 10 mL of H_2_O, and then refluxed for 2 h with stirring. At r.t., the reaction mixture was alkalinized with 6N NaOH and extracted with CH_2_Cl_2_; the organic layer was washed with H_2_O, then dried with anhydrous Na_2_SO_4_ and filtered. After evaporation, the oily residue was converted into the corresponding monohydrochloride with 1N ethanolic solution of HCl. Yield: 59%. M.p. 205–208 °C. ^1^H NMR (200 MHz, DMSO-*d*_6_): δ 8.14–7.48 (m, 4H, 2H pyr. and 7.78, s, N*H*_2_^+^superimposed, exchange with D_2_O), 6.97–6.61 (m, 4H, 2H pyr. and N*H_2_* exchange with D_2_O), 3.61–3.54 (m, 8H, 4C*H_2_* piperazine). ^13^C NMR (50 MHz, DMSO-*d*_6_): δ 162.3, 158.5, 149.6, 136.7, 115.6, 111.0, 46.7 (2C), 46.0 (2C). Anal. Calcd for C_10_H_15_N_5_·HCl: % C, 49.69; H, 6.67; N, 28.97. Found: C, 49.82; H, 6.76; N, 28.60.

### 3.2. In Vitro Biological Tests

#### 3.2.1. Screening of hTAAR1 Agonists by Means of Bioluminescence Resonance Energy Transfer (BRET) Technology

BRET screening was described in detail elsewhere [40]. HEK-293T (ATCC) cells were transiently сo-transfected with plasmids encoding *h*TAAR1 and a cAMP BRET biosensor using Lipofectamine^®^ (ThermoFisher) reagent, and then plated in 96-well plates (Corning) at 50 × 10^4^ cells per well. On the following day, culture medium was removed and 70 μL of phosphate-buffered saline containing calcium and magnesium was added to each well, followed by the addition of 10 μL 200 mM 3-isobutyl-1-methylxanthine solution (Sigma), and 10 μL 50 μM coelenterazine-h solution (Promega). All tested compounds were dissolved in DMSO to yield 10 mM stock solutions. After 10 min incubation, either 10 μL of vehicle or 10× of the concentrated solution of compound to be tested (10 μM final concentration) was added. Readings were collected using a Mithras LB943 multimodal plate reader (Berthold Technologies). The BRET signal is determined by calculating the ratio of the light emitted at 505 to 555 nm to the light emitted at 465 to 505 nm.

For active compounds, separate dose–response experiments were performed in order to calculate the EC_50_ values. Curves were fitted by applying non-linear regression models on GraphPad Prism 6 (GraphPad Software). Data are representative of 4 independent experiments and are expressed as means (errors in EC_50_ are within 10%).

#### 3.2.2. Cell Cytotoxicity Assay

Vero-76 cells (ATCC CRL 1587 *Cercopithecus Aethiops*) were seeded at an initial density of 4 × 10^5^ cells/mL in 24-well plates, in culture medium (Dulbecco’s Modified Eagle Medium (D-MEM) with L-glutamine, supplemented with fetal bovine serum (FBS), 0.025 g/L kanamycin). Cell cultures were then incubated at 37 °C in a humidified, 5% CO_2_ atmosphere in the absence or presence of serial dilutions of test compounds. Cell viability was determined after 48–96 h at 37 °C by the MTT staining method. The results are expressed as CC_50_, which is the concentration of compound necessary to inhibit cell growth by 50%. Each CC_50_ value is the mean and standard deviation of at least three separate experiments performed in duplicate.

### 3.3. Molecular Modelling Studies

#### 3.3.1. Ligand Preparation and Pharmacophore Analysis

All the compounds investigated herein through computational studies were built, parameterized (AM1 partial charges as in the calculation method) and energy minimized within MOE [41], following the same procedure we applied in our previous work [29,31]. Oxazolines were explored in terms of geometry and conformation energy by means of the systematic Conformational Search tool implemented in MOE, in order to be further exploited for pharmacophore analysis. Briefly, Systematic Conformational Search generates molecular conformations by systematically rotating bonds in a molecule by discrete increments. The purpose of Systematic Conformational Search is to generate a collection of reasonable molecular conformations, which may or may not be at local minima. A generated conformation is rejected if it contains two atoms whose mutual van der Waals energy exceeds a threshold (by default, 10 kcal/mol). This ensures that the output conformations contain no conformations with heavily overlapped atoms. Conformations generated by this method may be strained due to bonded interactions as well as some non-bonded strain. Then, a preliminary pharmacophore model has been developed taking into account the most potent oxazolines reported by Roche (pEC_50_ values > 7.00 M) including compounds **4b**, **5b**, **9b**–**11b**, **16b**, **18b**, **20b**–**23b**, **25b**, **29b** and **33b**–**37b** from the whole set **1b**–**37b**, while the remaining modest analogues were considered as an external set. The final model has been calculated using the pharmacophore search module implemented in the MOE software, starting from the alignment of the aforementioned oxazolines onto the most potent **20b**, taken as reference compound. The module pharmacophore consensus of this software generates a set of suggested features based on the exploited alignment of the selected oxazoline conformations. These chemical features are characterized by a position, radius and a type expression. The relevance of any feature when based on equal scores is assessed by secondary keys in the following order: radius, number of molecules, number of conformations, length of the expression and alphabetical sequence. This kind of analysis is described in the literature as a successful strategy for drug design and for virtual screening approaches. Accordingly, the computational part of pharmacophore modeling has significantly improved in reent years thanks to the availability of software packages, such as MOE software [42,43,44,45,46].

#### 3.3.2. Molecular Docking Studies

Docking calculations within the *h*TAAR1 receptor have been performed based on the in-house homology modelled *h*TAAR1, which has already been extensively exploited by us to explore the putative bioactive conformation and binding mode of different series of TAAR1-targeting compounds [29,31]. The reliability of our previous study, as well as of the binding site we discussed, was supported by mutagenesis experiments performed by Reese [47]. Herein, we proceeded with molecular docking studies at the same binding site, focusing on the newly synthesized compounds, **1**, **2**, **6** and **15**, featuring a higher potency trend within the amidine series. The reliability of the applied docking protocol was assessed by performing preliminary docking calculations on the oxazolines **20b**, **26b**, **27b** and **30b**, as reference compounds, and on β-phenylethylamine (β-PEA), as a pharmacological tool. On the whole, docking studies were performed by means of the DOCK tool implemented in MOE, choosing as a binding site the one identified in our previous studies [29,31]. The alpha triangle, as a placement algorithm, was selected. In this case, poses are generated by the superposition of ligand atom triplets and the triplets of receptor site points. The receptor site points are alpha sphere centers that represent the locations of tight packing. At each iteration, a random conformation is selected. A random triplet of ligand atoms and a random triplet of alpha sphere centers are used to determine the pose. The calculation of the enthalpy-based Affinity ΔG scoring function allowed us to score the generated thirty poses, while the induced fit method was applied to refine the previous poses and create the final ones. These were rescored based on the alpha HB methodology, which is focused on H-bonding estimation. 

This Affinity ΔG function estimates the enthalpic contribution to the free energy of binding using a linear function:

Δ*G* = *C*_hb_*f*_hb_ + *C*_ion_*f*_ion_ + *C*_mlig_*f*_mlig_ + *C*_hh_*f*_hh_ + *C*_hp_*f*_hp_ + *C*_aa_*f*_aa_

where the *f* terms fractionally count atomic contacts of specific types and the *C*_i_ terms are coefficients that weight the term contributions to the affinity estimate. The individual terms are shown below.
**Subscript****Description**hbInteractions between hydrogen bond donor–acceptor pairs. An optimistic view is taken; for example, two hydroxyl groups are assumed to interact in the most favorable wayionIonic interactions. A Coulomb-like term is used to evaluate the interactions between charged groups. This can contribute to or detract from binding affinitymligMetal ligation. Interactions between nitrogens/sulfurs and transition metals are assumed to be metal ligation interactionshhHydrophobic interactions, for example, between alkane carbons. These interactions are generally favorablehpInteractions between hydrophobic and polar atoms. These interactions are generally unfavorableaaAn interaction between any two atoms. This interaction is weak and generally favorable

The Induced Fit approach allows us to maintain flexible protein sidechains within the selected binding site, which are to be included in the refinement stage. The derived docking poses were prioritized by the score values of the lowest energy pose of the compounds docked to the protein structure, as shown below.
**S****The final score, which is the score of the last stage of refinement.****E_conf**The energy of the conformer. If there is a refinement stage, this is the energy calculated at the end of the refinement**E_place**Score from the placement stage**E_score1** **E_score2**Score from rescoring stages 1 and 2**E_refine**Score from the refinement stage, calculated to be the sum of the van der Waals electrostatics and solvation energies, under the Generalized Born solvation model (GB/VI)

#### 3.3.3. In Silico Evaluation of Pharmacokinetic and Toxicity Properties 

The prediction of parameters related to ADMET properties was performed by means of the Advanced Chemistry Development (ACD) Percepta platform 2015 (*v*14.0.0, www.acdlabs.com). Any ADMET descriptor was evaluated by Percepta, relying on the training libraries implemented in the software, which include several sets of molecules whose pharmacokinetic and toxicity behaviors have been experimentally determined.

## 4. Conclusions

The present work reports on the discovery of 1-amidino-4-phenylpiperazines as very promising *h*TAAR1 agonists, as a result of a combination strategy based on pharmacophore model studies and a scaffold simplification strategy for an in-house series of biguanide-based TAAR1 agonists. Interestingly, most of them showed a potent agonism activity at *h*TAAR1, comparing favorably with the nanomolar potency of the endogenous ligands and of notable compounds from La Roche. Our study has proven to be a valid strategy, allowing for the identification of the minimal structural requirements for an efficient *h*TAAR1 agonism behavior, with the amidino group as a basic moiety and a planar aromatic ring as the hydrophobic substituent, adequately spaced through the bifunctional piperazine ring and able to impose an adequate geometry to the molecule.

Additionally, they exhibited low cytotoxicity values; thus, the corresponding therapeutic indices were very high for the most effective *h*TAAR1 agonists (**15**, **2**, **9**, **16** and **6**). In other previous works, some of these compounds were proven to modulate the adrenergic system, both in the brain and in the periphery, an activity that was observed for other classes of TAAR1 ligands thus far identified.

Therefore, the present results prompt us to explore these 1-amidino-4-arylpiperazine derivatives at a later stage, with a view to assessing their TAAR1 selectivity profile over monoaminergic GPCRs, whose activation or downregulation might be correlated with the onset of unwanted side effects. Accordingly, this series deserves further structural improvements and more in-depth biological studies of their mechanism of action towards the design of more effective molecules endowed with well-suited drug-like properties. 

## Figures and Tables

**Table 1:** List of pharmacophore features shared by 80% of the oxazolines acting as *h*TAAR1 agonists, along with their ID, score, radius and expression parameters. Pharmacophore features: Aro = aromatic; Hyd = hydrophobic; Don = hydrogen bond donor; Acc = hydrogen bond acceptor.

ID	SCORE	RADIUS	EXPRESSION
**F1**	89%	1.96	Aro|Hyd
**F2**	83%	1.46	Hyd
**F3**	94%	1.36	Don2
**F4**	89%	1.08	Acc2
**F5**	89%	1.26	Don2
**F6**	100%	0.85	Acc
**F7**	100%	1.23	Don

**Table 2:** Functional activity at *h*TAAR1 receptor and cytotoxicity against Vero-76 of compounds **1**–**16**.

Compound	*h*TAAR1 Activity ^a^	*h*TAAR1 ^b^ EC_50_, nM	Vero-76 Cells ^c^ CC_50_, µM
**1**	71%	370	84 ± 5
**2**	100%	30	80 ± 5
**3**	90%	160	> 100
**4**	35%	>2000	90 ± 3
**5**	inactive	-	95 ± 3
**6**	64%	93	83 ± 5
**7**	32%	244	87 ± 4
**8**	inactive	-	88 ± 2
**9**	80%	64	>100
**10**	30%	>2000	74 ± 5
**11**	inactive	-	64 ± 2
**12**	inactive	-	78 ± 2
**13**	inactive	-	87 ± 5
**14**	inactive	-	94 ± 6
**15**	81%	20	82 ± 5
**16**	49%	71	89 ± 4
**TYR**	100%	66	-

^a^ As compared with 1 µM TYR (full agonism); ^b^ Data are representative of four independent experiments and are expressed as means (errors on EC_50_ are within 10%). ^c^ Compound concentration (µM) required to reduce the viability of Vero-76 cells by 50% as determined by the MTT method. The results are expressed as CC_50_±SEM of three separate experiments performed in duplicate.

**Table 3:** Calculated absorption, distribution, metabolism, excretion and toxicity properties (ADMET) descriptors related to absorption and distribution properties. The most promising amidine-containing derivative **15** has been highlighted in yellow, as well as the reference compounds β-PEA and RO5263397. The other most interesting analogues, **2**, **6**, **9** and **16**, are shown in light cyan. The only one derivative predicted as unable to pass the blood–brain barrier (BBB) is reported in gray.

Comp.	cLogP	LogBB ^a^	LogPS ^b^	HIA (%) ^c^	Vd (l/kg) ^d^	%PPB	LogKa ^HSA^	%F (oral)
**β-PEA**	1.56	0.19	−2.4	99	3.0	34.79	2.78	91.1
**1**	0.31	−0.08	−3.6	50	1.2	33.14	2.84	28.9
**2**	0.71	−0.03	−3.4	58	1.4	33.91	2.85	35.7
**3**	1.15	−0.04	−3.2	74	1.3	48.25	3.19	49.7
**4**	0.99	−0.08	−3.2	68	1.5	49.24	3.11	44.5
**5**	0.81	−0.13	−3.3	61	1.4	49.93	3.15	38.5
**6**	0.33	−0.08	−3.7	45	1.2	33.69	2.90	27.1
**7**	0.36	−0.08	−3.7	46	1.3	33.00	2.82	27.1
**8**	0.16	−0.09	−3.9	41	1.1	31.42	2.86	23.2
**9**	1.26	−0.05	−3.1	77	1.6	52.86	3.34	54.1
**10**	−0.31	−0.13	−4.0	43	1.0	32.87	2.68	25.2
**11**	−0.43	−0.09	−4.1	42	0.7	25.03	2.49	25.2
**12**	0.04	−0.04	−3.8	40	1.2	21.76	2.51	22.9
**13**	0.45	−0.03	−3.5	53	1.2	29.16	2.97	32.3
**14**	0.37	−0.01	−3.5	51	1.1	23.28	2.51	30.6
**15**	1.72	0.13	−2.9	92	1.5	50.18	3.57	66.9
**16**	1.88	0.19	−2.8	95	1.6	50.85	3.52	76.9
**RO5263397**	1.85	0.35	−2.0	100	2.4	27.57	3.12	99.0

^a^ Extent of brain penetration based on ratio of total drug concentrations in tissue and plasma at steady-state conditions; ^b^ Rate of brain penetration. The permeability–surface area product (PS) is derived from the kinetic equation of capillary transport; ^c^ human intestinal absorption (HIA) is expressed as percentage of the molecule able to pass through the intestinal membrane; ^d^ prediction of volume of distribution (Vd) of the compound in the body.

**Table 4:** Calculated ADMET descriptors related to metabolism, excretion and toxicity properties. The most promising amidine-containing derivative **15** has been highlighted in yellow, as well as the reference compounds β-PEA and RO5263397. The other most interesting analogues, **2**, **6**, **9** and **16**, are shown in light cyan.

Comp.	hERG Inhibitor		Endocrine System Disruption ^a^		CYP3A4 and CYP2D6	
	Inhibitor	Reliability Index (R.I.)	LogRBA > −3 (R.I. ≥ 0.3)	LogRBA > 0 (R.I. ≥ 0.3)	Inhibitor < 10 mM (R.I. > 0.3)	Substrate (R.I. ≥ 0.3)
**β-PEA**	0.06	0.40	No binder	No binder	0.02	>50%
**1**	0.07	0.34	No binder	No binder	0.01	>50%
**2**	0.08	0.35	No binder	No binder	0.01	>50%
**3**	0.11	0.29	No binder	No binder	0.01	>50%
**4**	0.11	0.29	No binder	No binder	0.01	>50%
**5**	0.15	0.29	No binder	No binder	0.01	>50%
**6**	0.10	0.29	No binder	No binder	0.02	>50%
**7**	0.09	0.29	No binder	No binder	0.02	>50%
**8**	0.15	0.22	No binder	No binder	0.02	>50%
**9**	0.43	0.56	No binder	No binder	0.01	>50%
**10**	0.07	0.35	No binder	No binder	0.01	>50%
**11**	0.05	0.33	No binder	No binder	0.02	>50%
**12**	0.07	0.30	No binder	No binder	0.01	>50%
**13**	0.12	0.25	No binder	No binder	0.01	>50%
**14**	0.14	0.27	No binder	No binder	0.01	>50%
**15**	0.21	0.24	No binder	No binder	0.01	>50%
**16**	0.27	0.30	No binder	No binder	0.01	>50%
**RO5263397**	0.06	0.36	No binder	No binder	0.01	33%

^a^ RBA: relative binding affinity with respect to that of estradiol. Compounds showing LogRBA > 0 are classified as strong estrogen binders, while those showing LogRBA < −3 are considered as non-binders. (RI: reliability index. Borderline-allowed values for reliability parameter are ≥ 0.3; the most predictive fall in the range 0.50–1.0).

## Supplementary Materials

The following are available online at https://www.mdpi.com/1424-8247/13/11/391/s1: Chemical structure and biological activity of 2-aminooxazolines (**1b**–**37b**) (Table S1); Table of scoring functions for the selected docking poses of the discussed *h*TAAR1 agonists (Table S2); Docking positioning at the *h*TAAR1 putative binding site of the oxazoline derivatives **20b** (Figure S1) and **30b** (Figure S2), of the amidine derivatives **1** (Figure S3), **2** and **15** (Figure S4); Analysis of the ChEMBL database depending on different similarity thresholds to the present 1-amidino-4-phenylpiperazines **1**–**16** (Table S3); ^1^H and ^13^C NMR spectra of compounds **3**, **6**, **7**, **9**, **12**, **15** and **16**.
