# Supplementary material for: Novel 1-Amidino-4-Phenylpiperazines as Potent Agonists at Human TAAR1 Receptor: Rational Design, Synthesis, Biological Evaluation and Molecular Docking Studies
Source: Pharmaceuticals (Basel). 2020 Nov 14;13(11):391. doi: 10.3390/ph13110391 (PMC7697893; doi:10.3390/ph13110391)

SUPPLEMENTARY MATERIALS

Novel 1-amidino-4-phenylpiperazines as potent agonists at human TAAR1 receptor: rational design, synthesis, biological evaluation and molecular docking studies

Valeria Francesconi [1], Elena Cichero [1,*], Evgeny V. Kanov [2], Erik Laurini [3], Sabrina Pricl [3,4], Raul R. Gainetdinov [2,5] and Michele Tonelli [1,*]

Table S1. Chemical structure and biological activity of 2-aminooxazolines (**1b-37b**) acting as *h*TAAR1 agonists previously exploited for QSAR analyses [30].

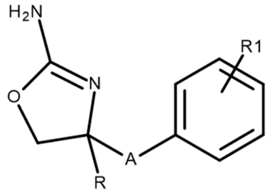

**Comp.**	**R**	**A**	**R1**	**Configuration**	**pEC$_{50}$ (M)**
**1b**	H	-CH_2-	2-Cl	*S*	6.81
**2b**	H	-CH_2-	H	*S*	6.48
**3b**	H	-CH_2-	H	*R*	5.54
**4b**	H	-CH_2CH_2-	3-Cl	*S*	7.74
**5b**	H	-CH_2CH_2-	H	*S*	7.57
**6b**	CH_3	-CH_2CH_2-	3-Cl	*S*	6.48
**7b**	H	-CH_2O-	3-Cl	*S*	6.57
**8b**	H	-CH_2NH-	3-Cl	*S*	6.24
**9b**	H	-CH_2NCH_3-	3-Cl	*S*	7.57
**10b**	H	-$CH_2NC_2H_5$-	3-Cl	*S*	7.54
**11b**	H	-$CH_2NC_2H_5$-	H	*S*	7.23
**12b**	H	-CH_2N^iPr-	H	*S*	6.85
**13b**	H	-$CH_2NC_2H_5$-	H	*R*	6.64

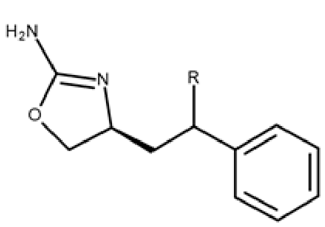

**Comp.**	**R**	**pEC$_{50}$ (M)**
**14b**	(*S*)-CH_3	5.81
**15b**	(*R*)-CH_3	6.14
**16b**	(*S*)-C_2H_5	7.74
**17b**	(*R*)-C_2H_5	5.65

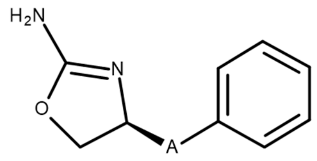

Comp.	A	pEC50 (M)
**18b**	$-CH_2CH_2CH_2-$	7.57
**19b**	$-CH_2OCH_2-$	6.44
**20b**	$-CH_2CH_2O-$	8.05

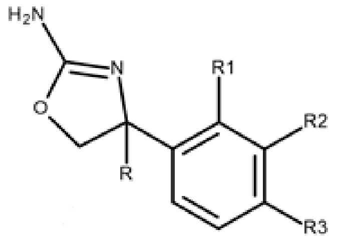

Comp.	R	R1	R2	R3	Configuration	pEC50 (M)
**21b**	H	H	H	H	*S*	7.17
**22b**	H	Cl	H	H	*S*	7.64
**23b**	H	H	Cl	H	*S*	7.68
**24b**	H	H	H	Cl	*S*	6.84
**25b**	H	H	Cl	Cl	*S*	7.51
**26b**	H	H	H	Br	*S*	6.82
**27b**	H	H	H	Br	*R*	5.00
**28b**	CH_3	Cl	H	H	*S*	6.78
**29b**	CH_3	H	H	Br	*S*	7.39
**30b**	H	H	H	Ph	*S*	5.57
**31b**	H	H	OPh	H	*R/S*	5.71
**32b**	H	H	F	H	*S*	6.31
**33b**	H	CH_3	H	H	*S*	7.17
**34b**	H	CH_3	H	Cl	*S*	7.96
**35b**	H	CH_2CH_3	H	Cl	*S*	7.59
**36b**	H	*c*Pr	H	Cl	*S*	7.92
**37b**	H	CH_3	F	H	*S*	7.77

Table S2. Table of scoring functions for the selected docking poses of the discussed *h*TAAR1 agonists.

**Compound**	**S**	**E_conf**	**E_place**	**E_score1**	**E_refine**	**E_score2**
**20b**	-99.1078	-109.1025	-10.3802	-5.6912	-33.6487	-99.1078
**26b**	-96.2118	-108.1425	-10.5221	-5.1112	-31.4564	-96.2118
**27b**	-87.6534	-102.8943	-8.9768	-6.7823	-28.8635	-87.6534
**30b**	-84.7643	-107.8932	-9.0065	-7.6523	-28.6522	-84.7643
**1**	-85.7222	-319.0324	-11.0525	-5.1549	-22.5136	-85.7222
**2**	-94.6754	-310.7677	-11.0066	-6.4366	-28.9887	-94.6754
**6**	-94.5612	-280.4532	-12.6753	-5.7821	-27.9911	-94.5612
**15**	-95.0332	-281.8556	-12.7560	-6.2340	-29.0032	-95.0332
**PEA**	-95.8775	-109.6754	-9.6544	-4.7865	-27.7765	-95.8775

Figure S1. Docking positioning of the oxazoline derivative **20b** (C atom, yellow) and β-**PEA** (C atom; green) at the *h*TAAR1 putative binding site. Ligands are shown in sticks. The most relevant residues are labelled.

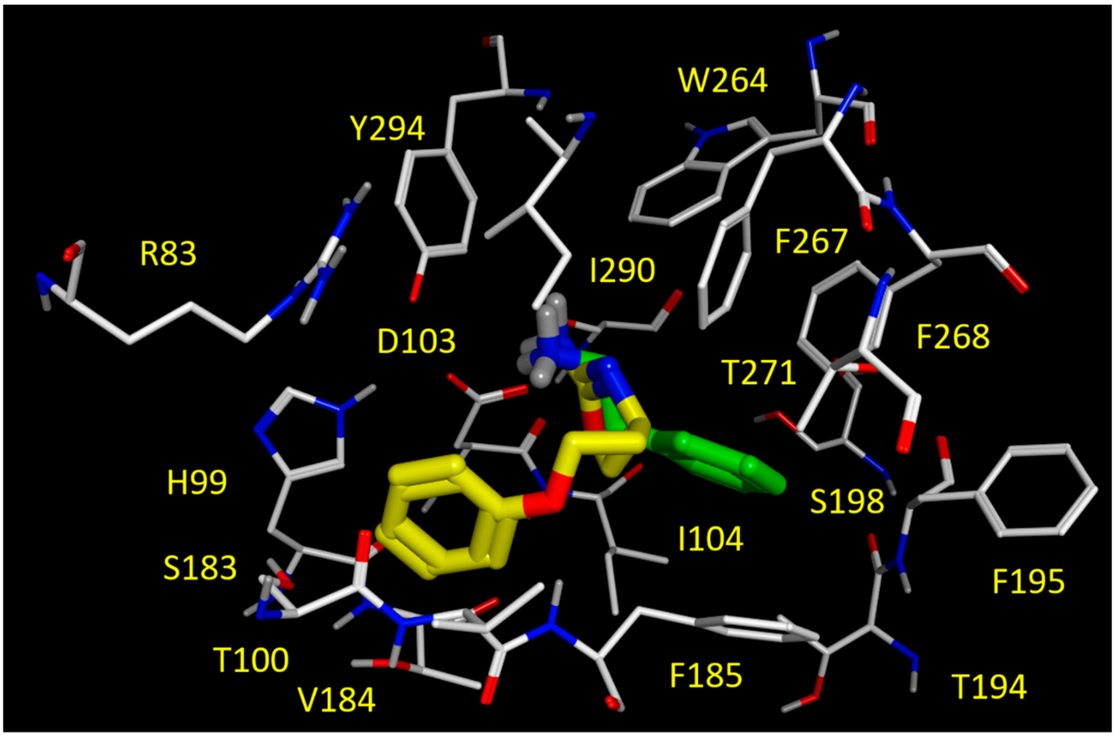

Figure S2. Docking positioning of the oxazoline derivative **30b** at the *h*TAAR1 putative binding site is shown as ligplot. The most relevant residues are labelled, being represented the most relevant H-bonds experienced by the agonist.

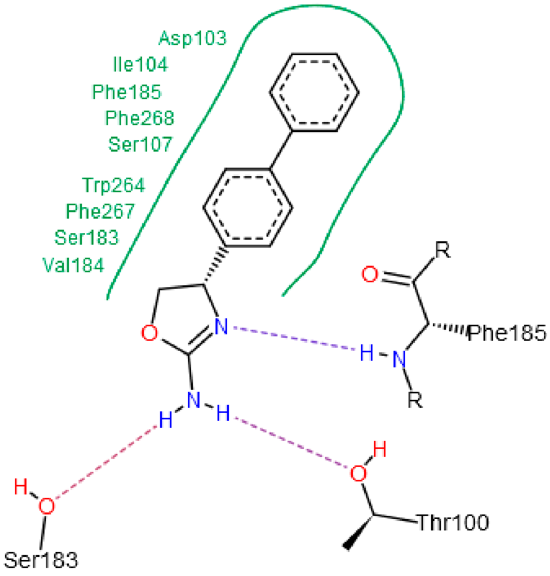

Figure S3. Docking positioning of the amidine derivative **1** (C atom, yellow) and **PEA** (C atom; green) at the *h*TAAR1 putative binding site. Ligands are shown in sticks. The most relevant residues are labelled.

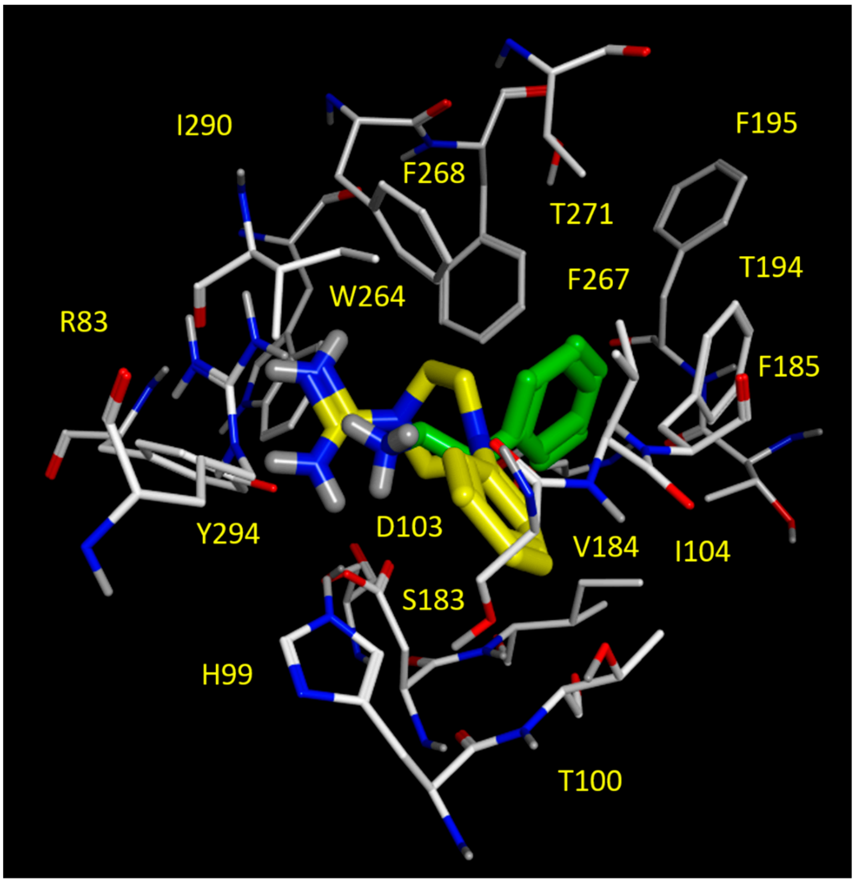

Figure S4. Docking positioning of the amidine derivative **2** (C atom, orange) and **15** (C atom; magenta) at the *h*TAAR1 putative binding site. Ligands are shown in sticks. The most relevant residues are labelled.

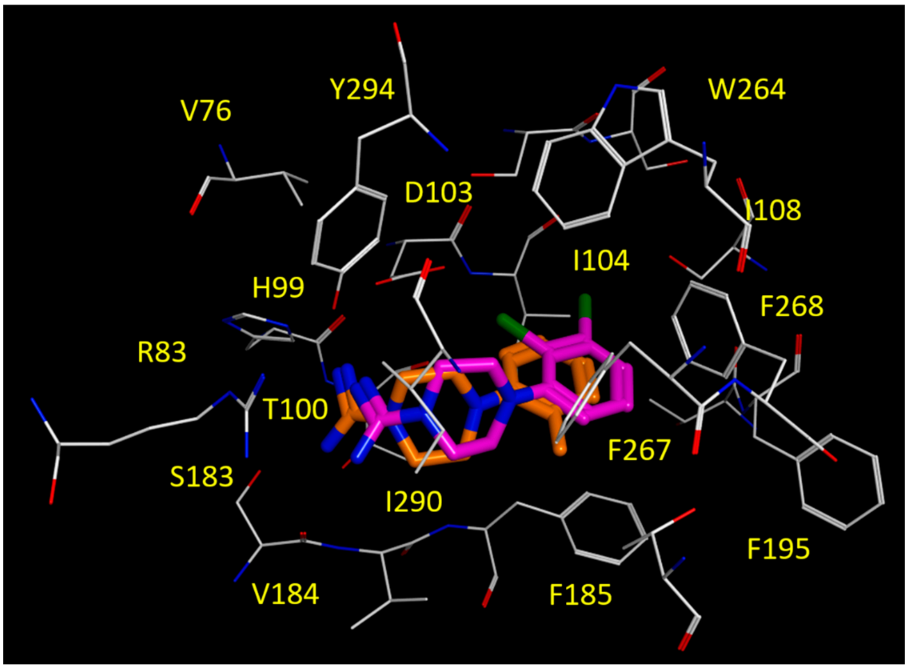

Table S3. Analysis of the ChEMBL database depending on different similarity thresholds to the present 1-amidino-4-phenylpiperazines **1-16**.

Compounds	First similar structures from ChEMBL database
	Similarity 67.74%<br>Similarity 66.66%
	Threshold: 60%<br>no records were found

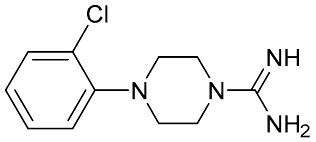 **3**	Similarity 71.42% 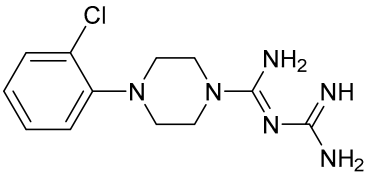 CHEMBL4172220 *
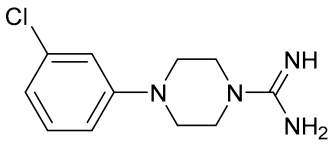 **4**	Similarity 73.68% 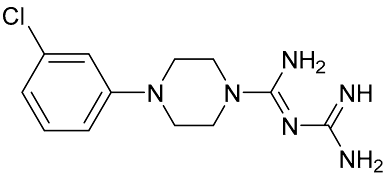 CHEMBL4163065 *
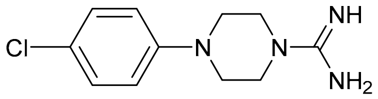 **5**	Similarity 100.0% 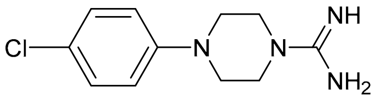 CHEMBL1215544 Similarity 70.59% 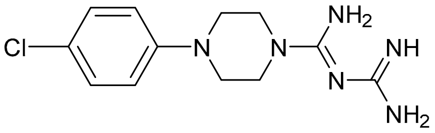 CHEMBL4173769 *
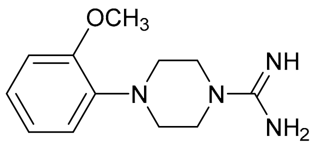 **6**	Similarity 74.36% 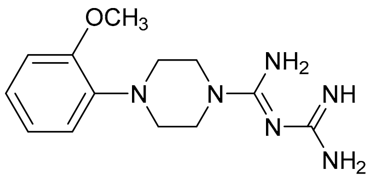 CHEMBL4169509 *
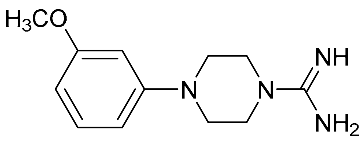 **7**	Similarity 75.0% 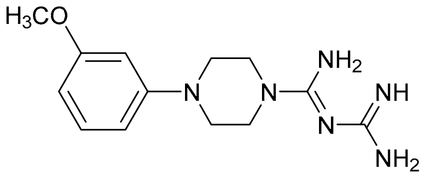 CHEMBL4177366 *

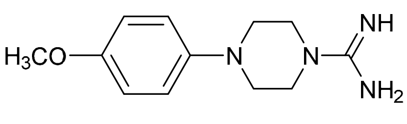 **8**	Similarity 72.97 % 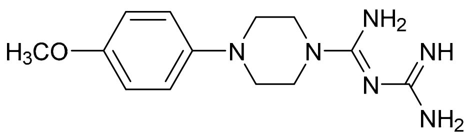 CHEMBL4172905 *
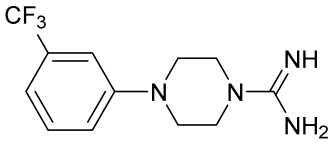 **9**	Similarity 75.61% 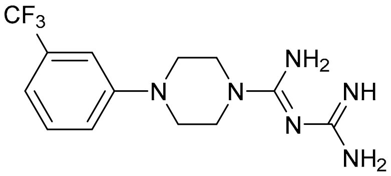 CHEMBL4161604 *
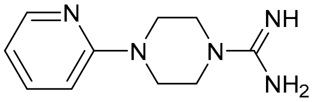 **10**	Similarity 72.97% 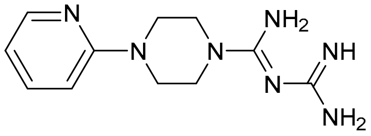 CHEMBL4162232 Similarity 71.43% 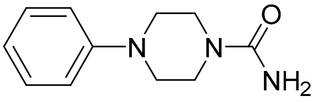 CHEMBL296963
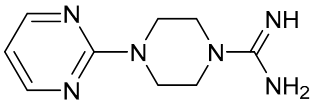 **11**	Similarity 68.0% 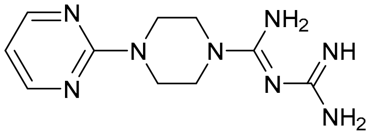 CHEMBL4162640
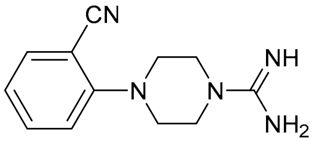 **12**	Threshold: 60% no records were found
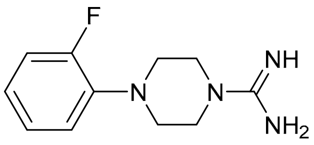 **13**	Similarity 59.46% 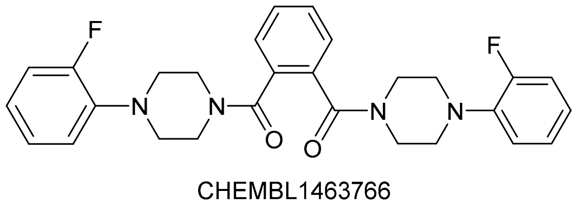 CHEMBL1463766

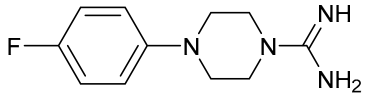<br>**14**	Similarity 68.75 %<br>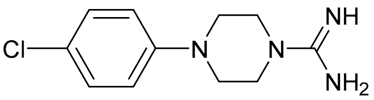 CHEMBL1215544
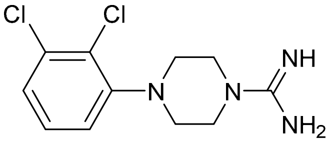<br>**15**	Threshold: 70%<br>no records were found
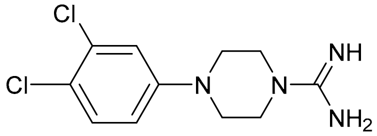<br>**16**	Threshold: 70%<br>no records were found

* Piperazino-based biguanides (SET2) previously synthesized by the Authors; see REF 31 (CHEMBL4145505): Guariento, S.; Tonelli, M.; Espinoza, S.; Gerasimov, A.S.; Gainetdinov, R.R.; Cichero, E. Rational design, chemical synthesis and biological evaluation of novel biguanides exploring species-specificity responsiveness of TAAR1 agonists. *Eur. J. Med. Chem*. **2018**, *146*, 146 171-184

^{1}H and ^{13}C NMR spectra of 4-(2-Chlorophenyl)piperazine-1-carboximidamide hydrochloride (**3**)

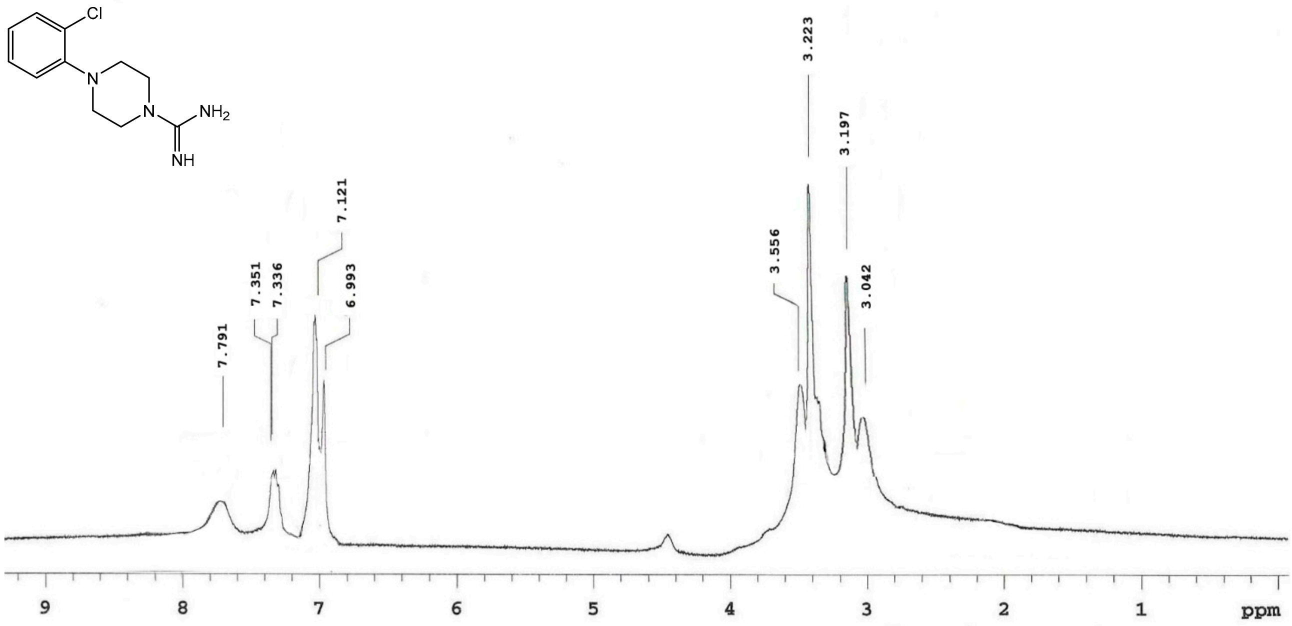

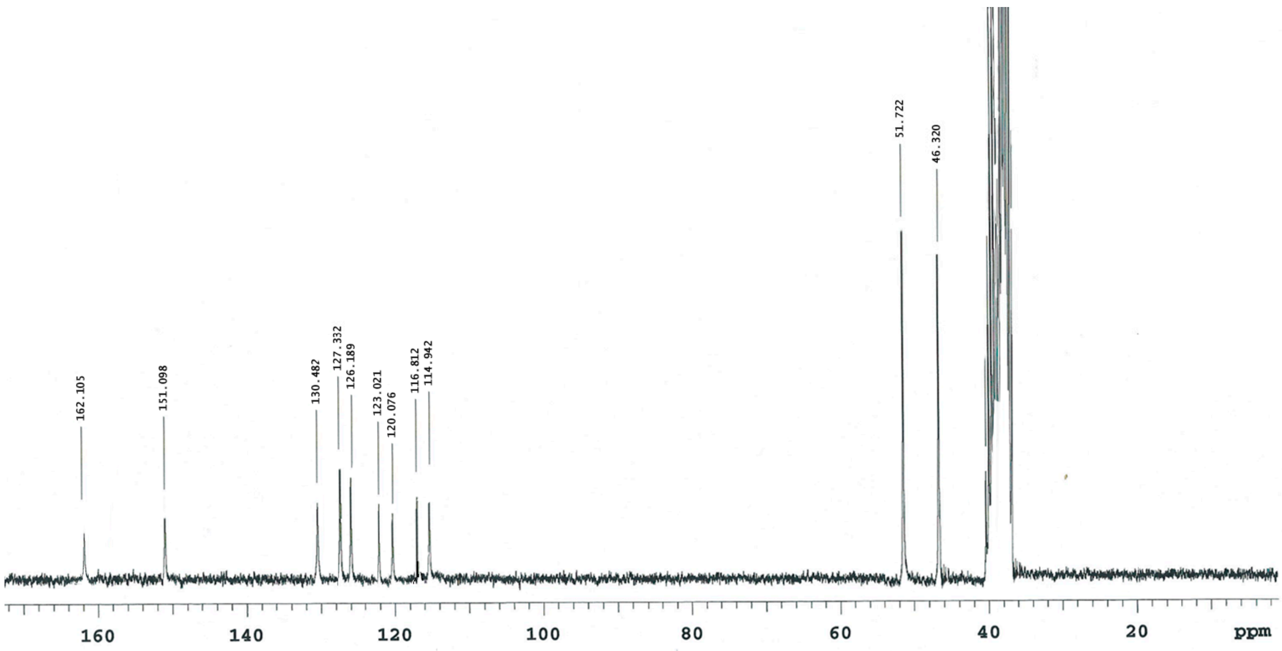

^{1}H and ^{13}C NMR spectra of 4-(2-Methoxyphenyl)piperazine-1-carboximidamide hydrochloride (**6**)

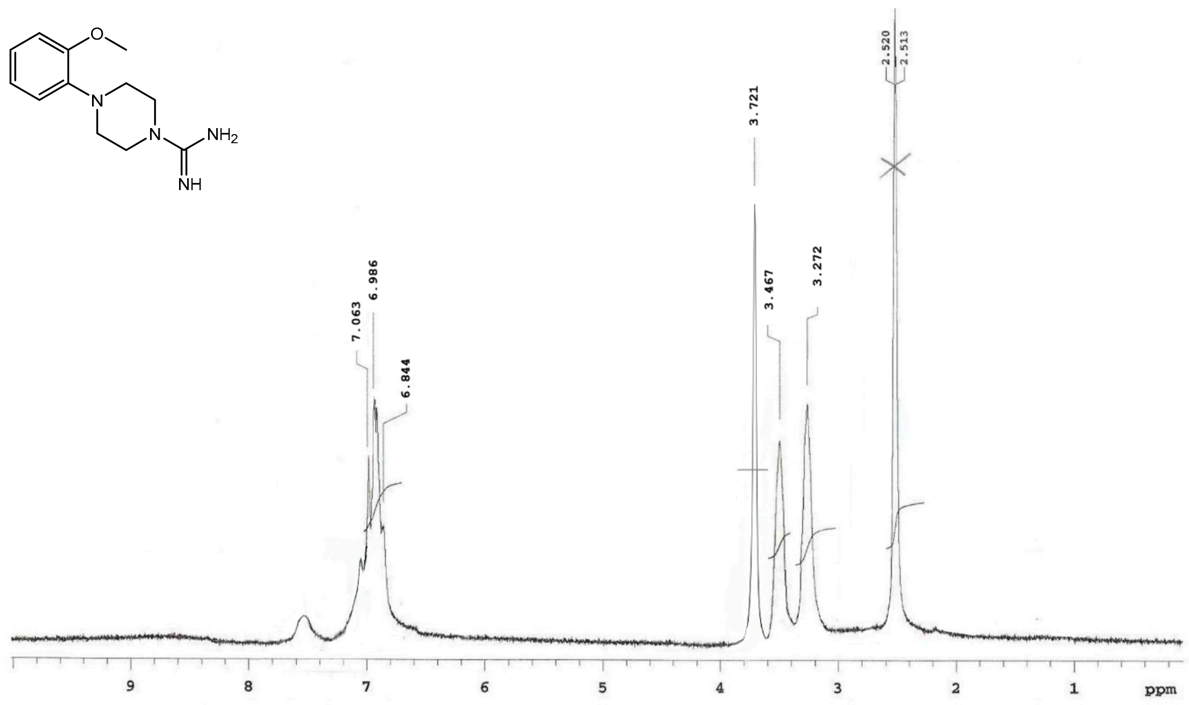

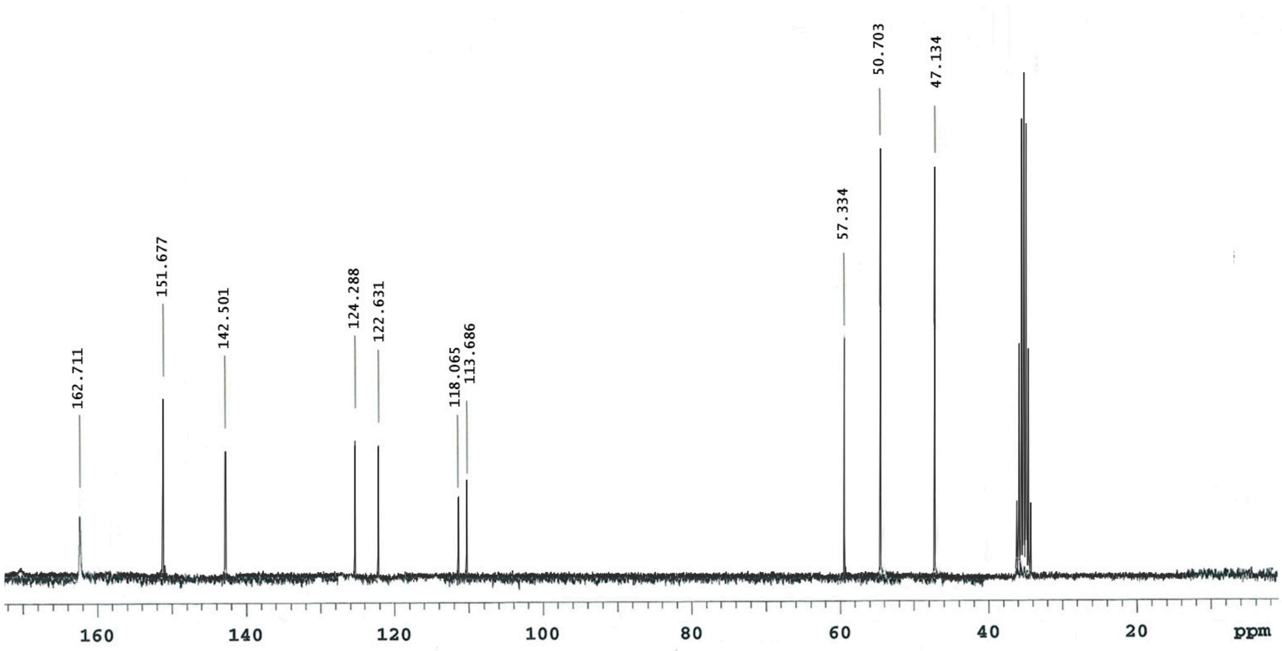

^{1}H and ^{13}C NMR spectra of 4-(3-Methoxyphenyl)piperazine-1-carboximidamide hydrochloride (**7**)

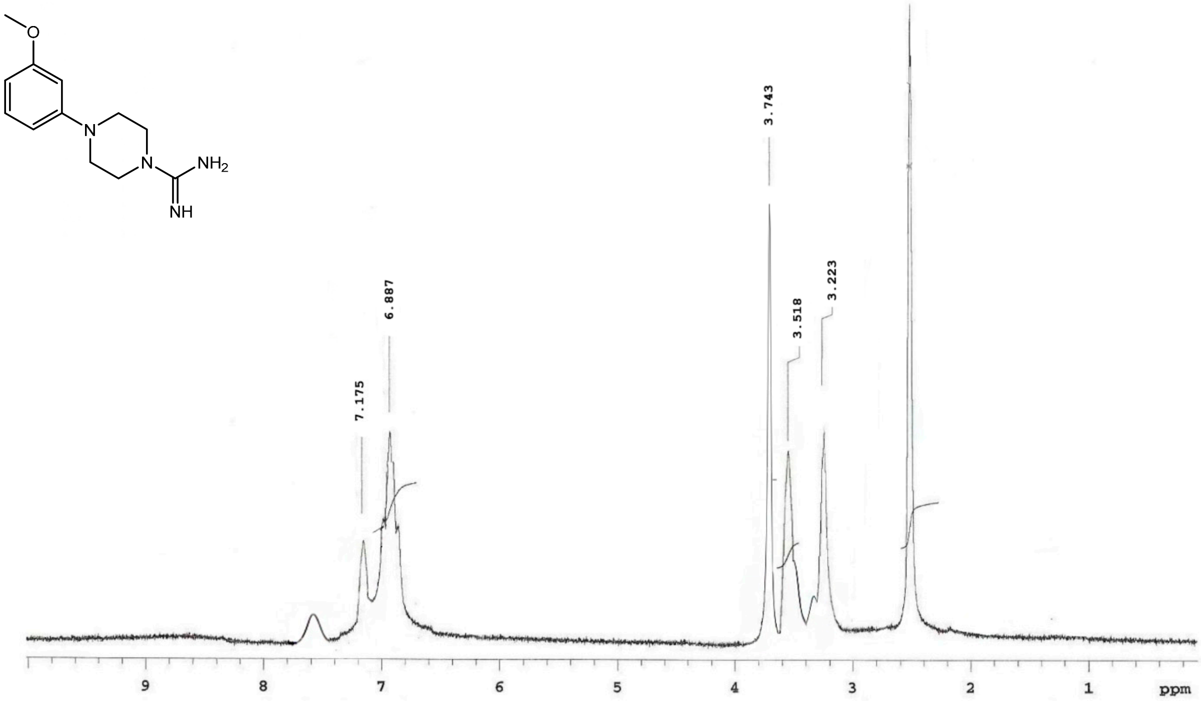

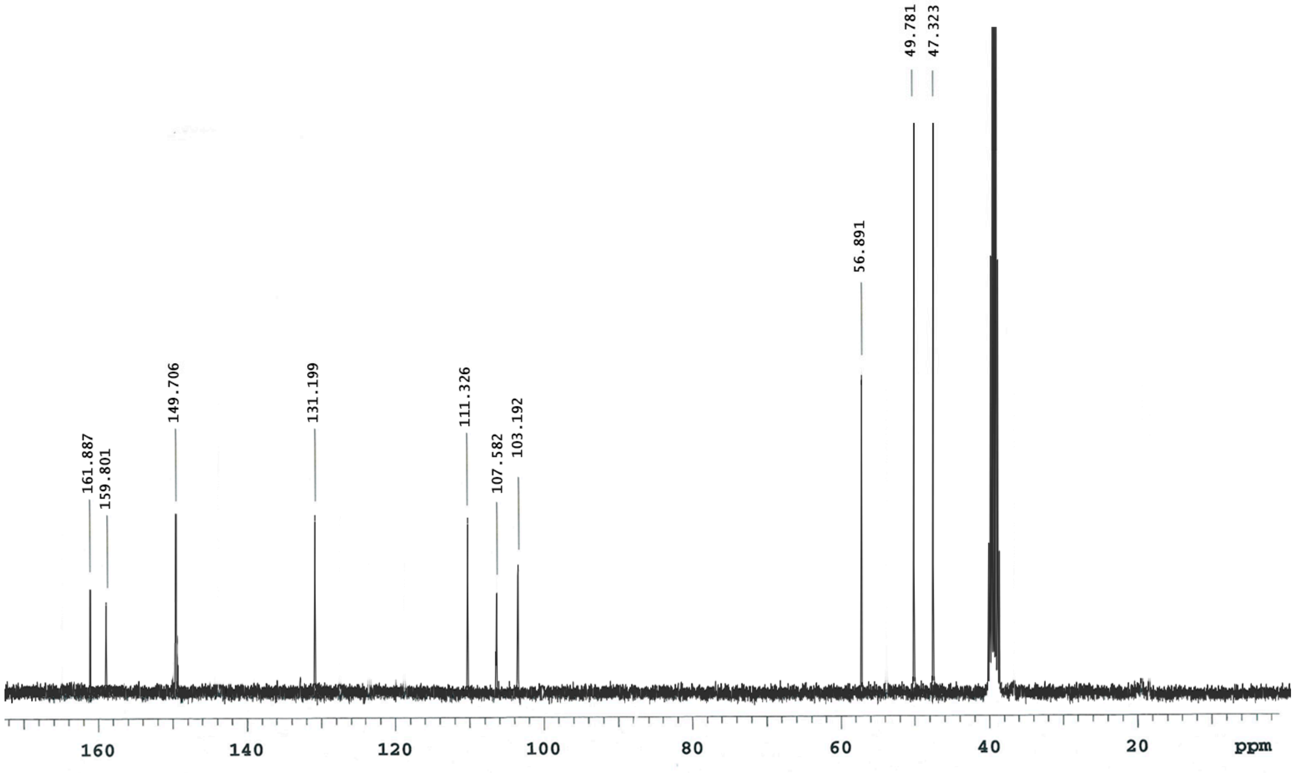

^{1}H and ^{13}C NMR spectra of 4-(3-trifluorophenyl)piperazine-1-carboximidamide hydrochloride (**9**)

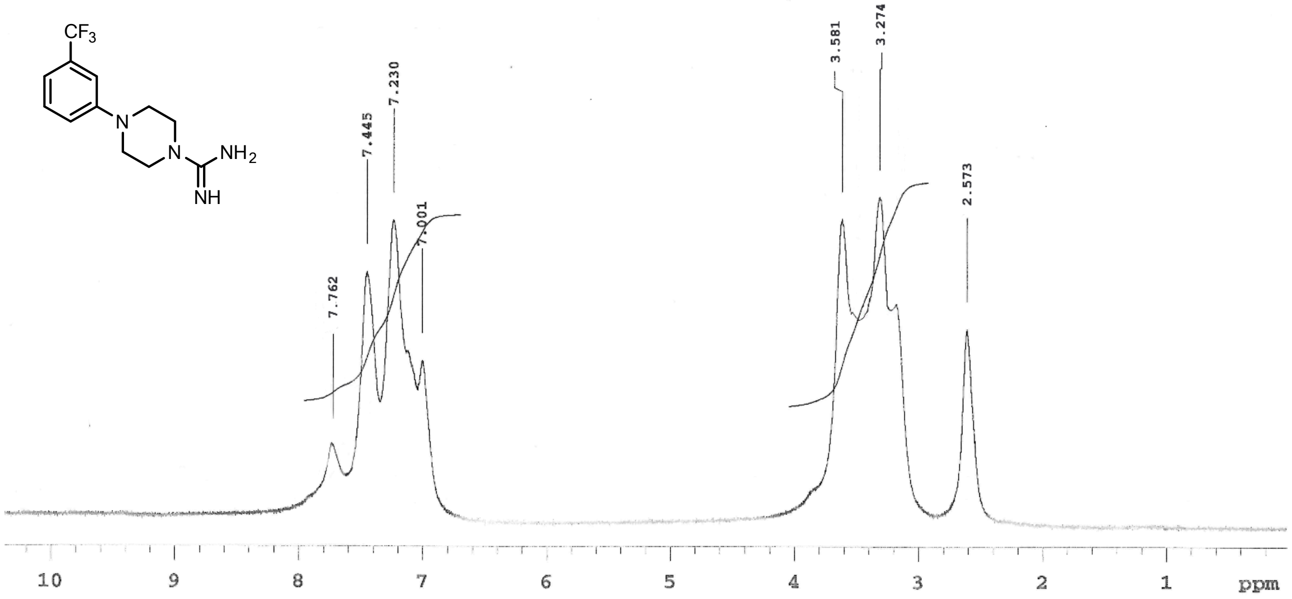

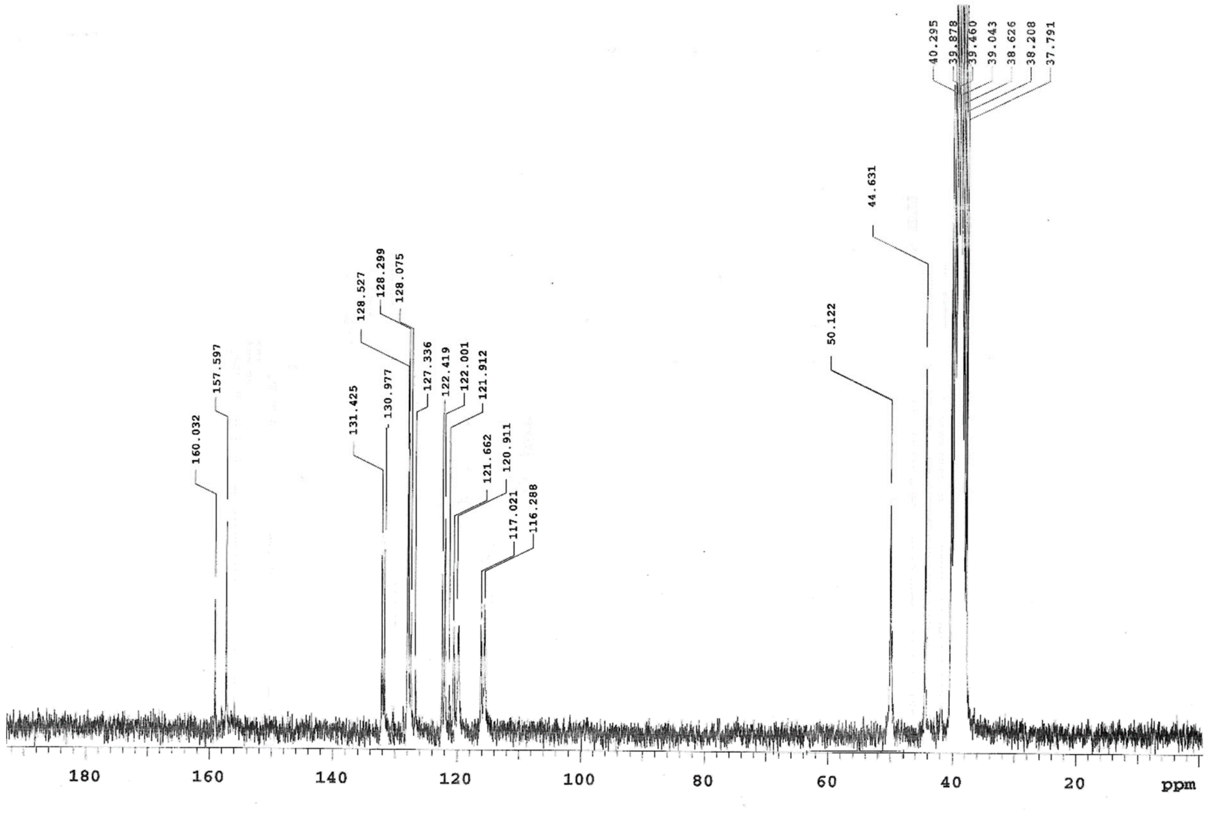

^{1}H and ^{13}C NMR spectra of 4-(2-cyanophenyl)piperazine-1-carboximidamide hydrochloride (**12**)

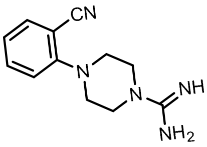

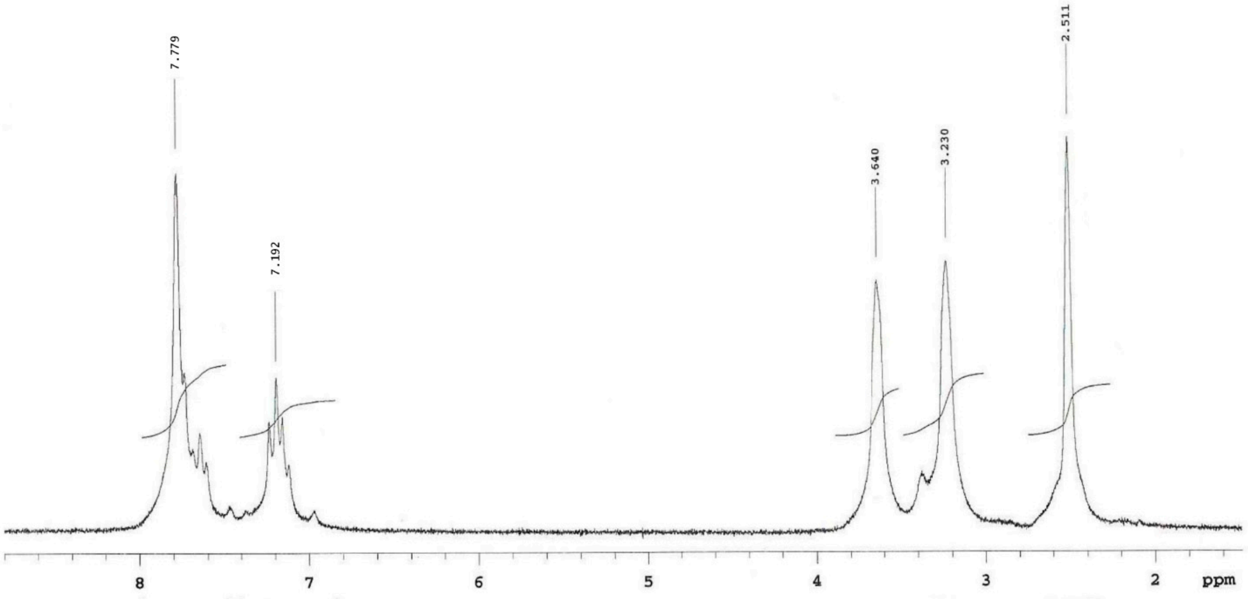

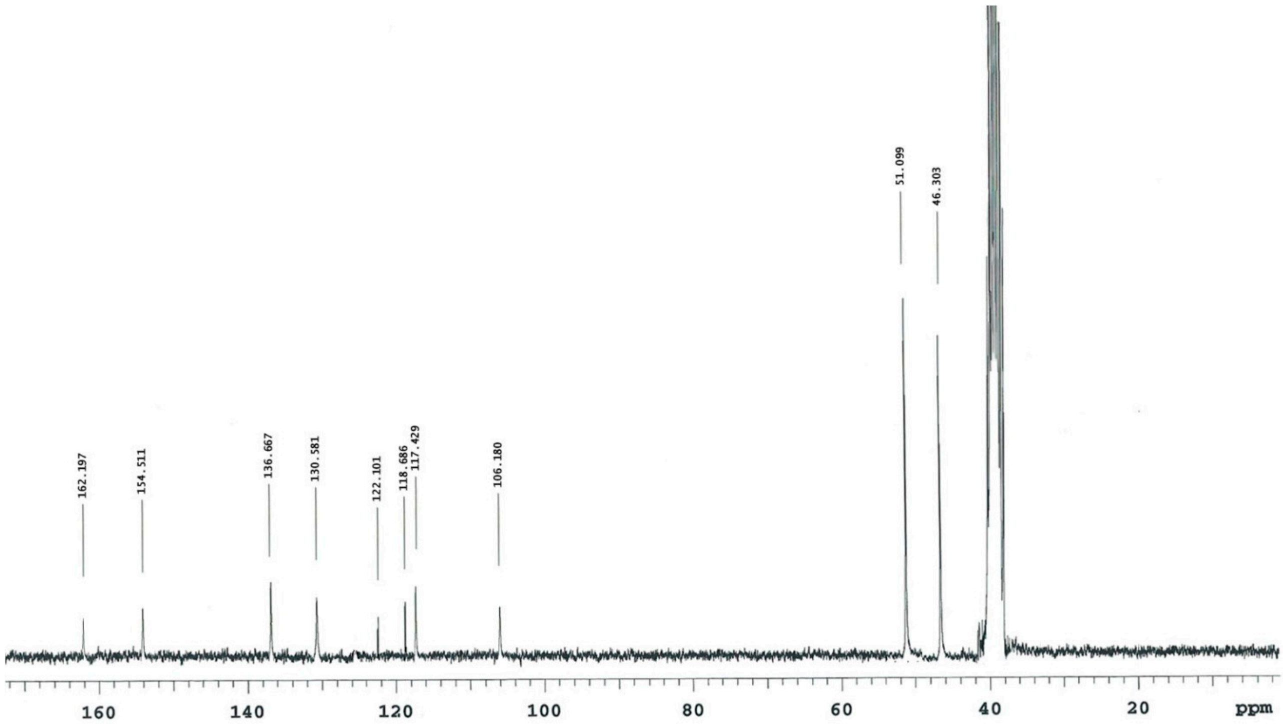

^{1}H and ^{13}C NMR spectra of 4-(2,3-Dichlorophenyl)piperazine-1-carboximidamide hydrochloride (**15**)

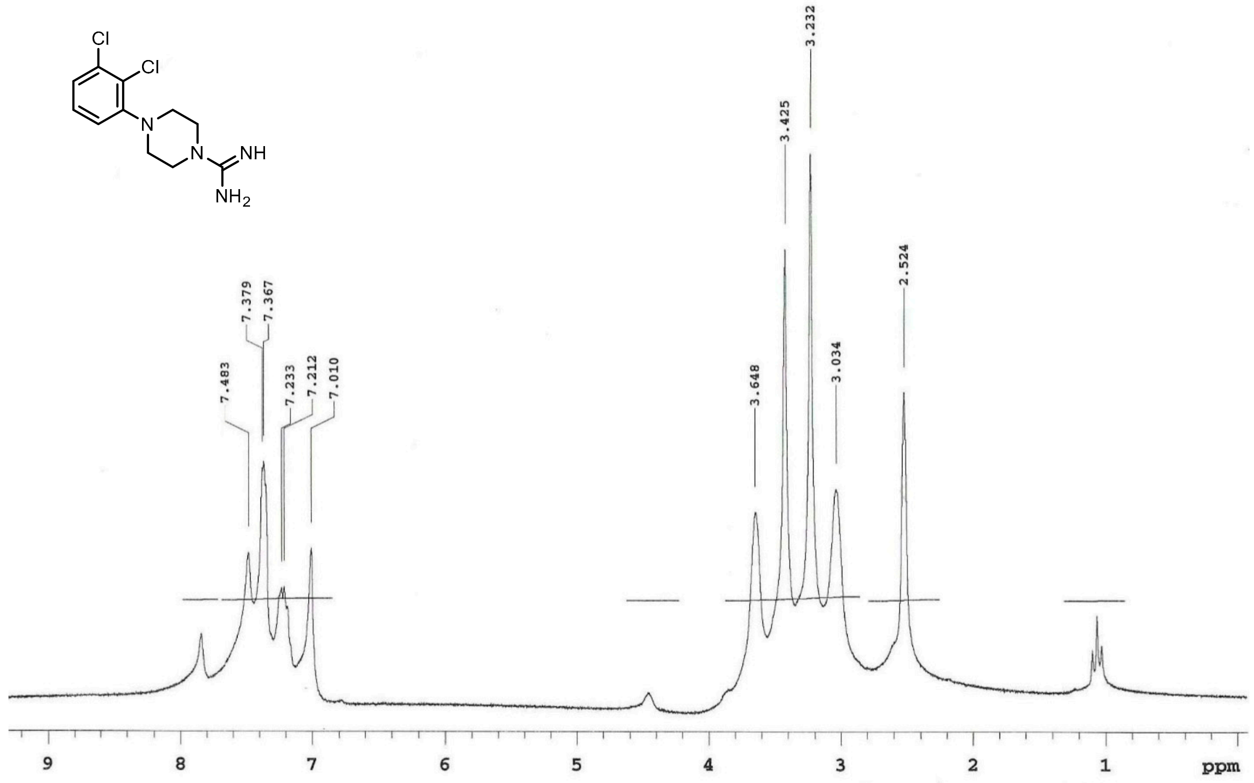

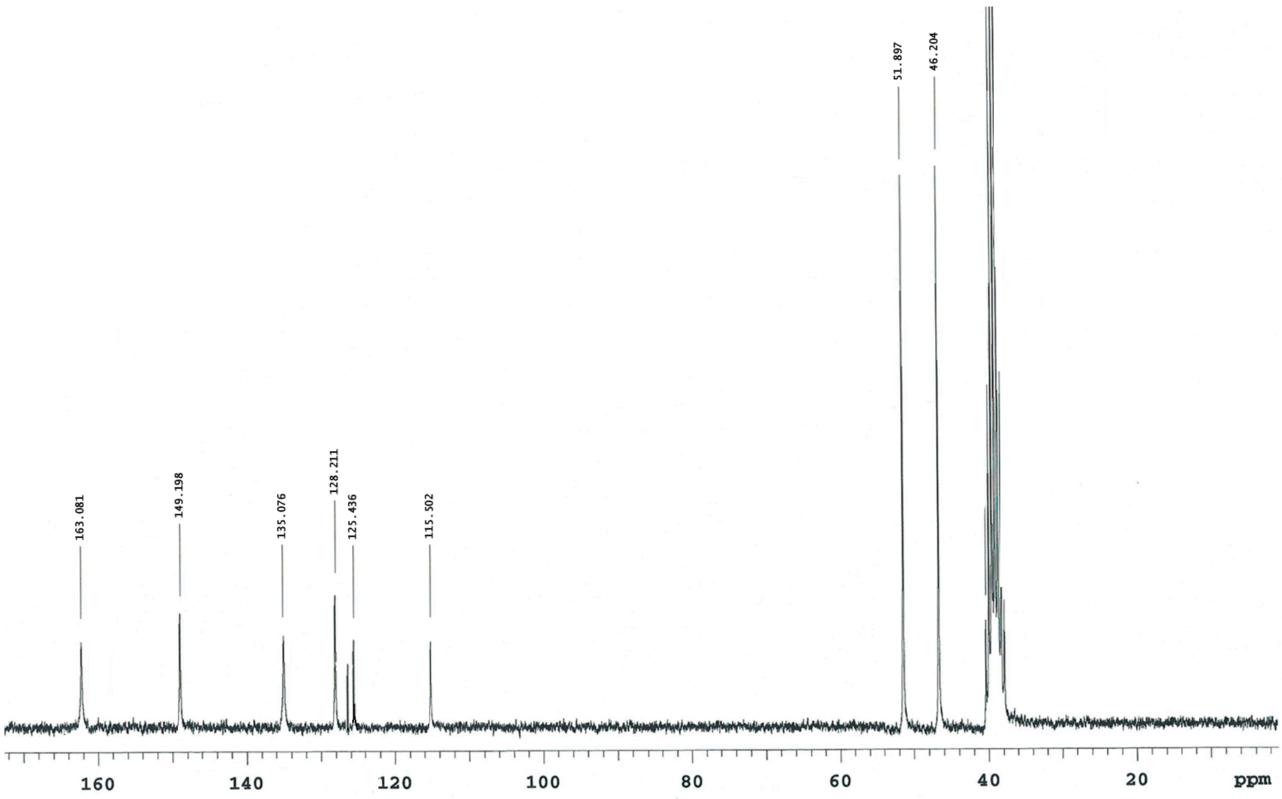

^{1}H and ^{13}C NMR spectra of 4-(3,4-Dichlorophenyl)piperazine-1-carboximidamide hydrochloride (**16**)

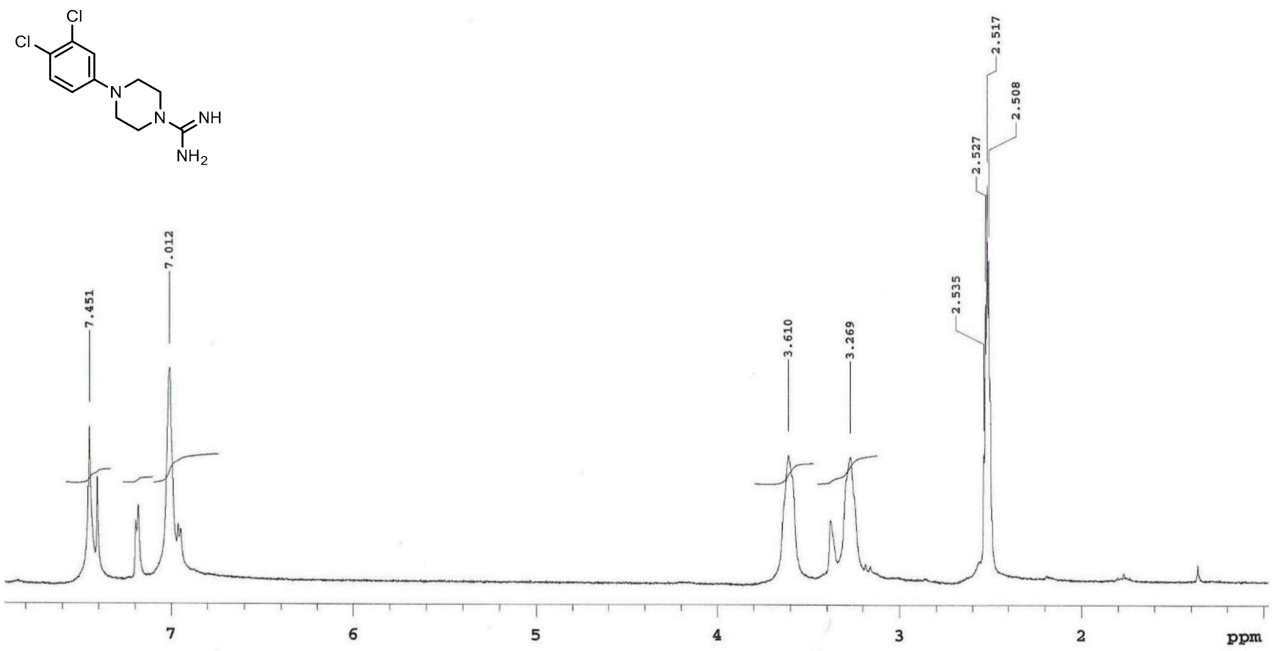

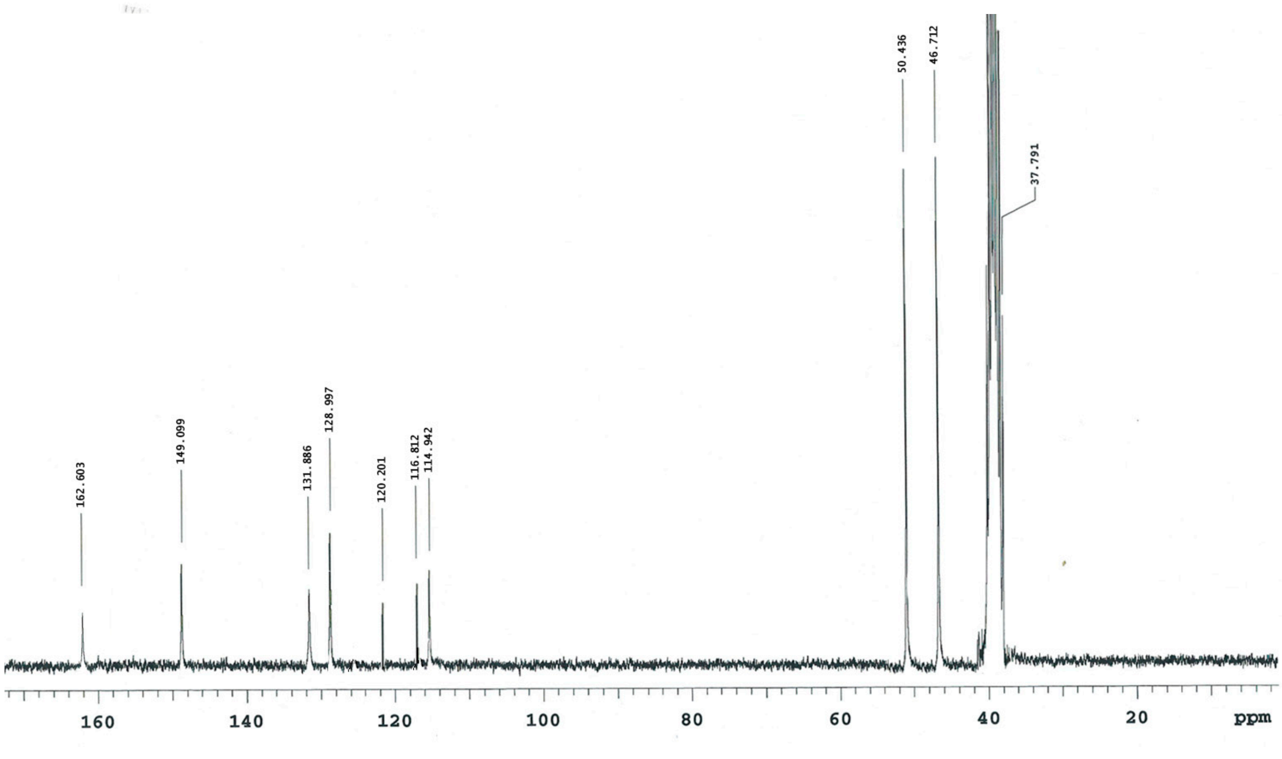

Supplement: Supplementary file 1 [file pharmaceuticals-13-00391-s001.pdf]
